# Linking microscopy to diffusion MRI with degenerate biophysical models: An application of the Bayesian EstimatioN of CHange (BENCH) framework

**DOI:** 10.1162/IMAG.a.85

**Published:** 2025-07-24

**Authors:** Daniel Z.L. Kor, Hossein Rafipoor, Istvan N. Huszar, Adele Smart, Greg Daubney, Saad Jbabdi, Michiel Cottaar, Karla L. Miller, Amy F.D. Howard

**Affiliations:** Wellcome Centre for Integrative Neuroimaging, FMRIB, Nuffield Department of Clinical Neurosciences, University of Oxford, Oxford, United Kingdom; Academic Unit of Neuropathology, Nuffield Department of Clinical Neurosciences, University of Oxford, Oxford, United Kingdom; Wellcome Centre for Integrative Neuroimaging, Experimental Psychology, Medical Sciences Division, University of Oxford, Oxford, United Kingdom; Department of Bioengineering, Imperial College London, London, United Kingdom

**Keywords:** MRI-microscopy, biophysical modelling, diffusion MRI, parameter inference, microstructure

## Abstract

Biophysical modelling of diffusion MRI (dMRI) is used to non-invasively estimate microstructural features of tissue, particularly in the brain. However, meaningful description of tissue requires many unknown parameters, resulting in a model that is often ill-posed. The Bayesian EstimatioN of CHange (BENCH) framework was specifically designed to circumvent parameter fitting for ill-conditioned models when one is simply interested in interpreting signal changes related to some variable of interest. To understand the biological underpinning of some observed change in MR signal between different conditions, BENCH predicts which model parameter, or combination of parameters, best explains the observed change, without having to invert the model. BENCH has been previously used to identify which biophysical parameters could explain group-wise dMRI signal differences (e.g., patients vs. controls); here, we adapt BENCH to interpret dMRI signal changes related to continuous variables. We investigate how parameters from the dMRI standard model of white matter, with an additional sphere compartment to represent glial cell bodies, relate to tissue microstructure quantified from histology. We validate BENCH using synthetic dMRI data from numerical simulations. We then apply it to ex-vivo macaque brain data with dMRI and microscopy metrics of glial density, axonal density, and axonal dispersion in the same brain. We found that (i) increases in myelin density are primarily associated with an increased intra-axonal volume fraction and (ii) changes in the orientation dispersion derived from myelin microscopy are linked to variations in the orientation dispersion index. Finally, we found that the dMRI signal is sensitive to changes in glial cell load in the brain white matter, though no single parameter in the extended standard model was able to explain this observed signal change.

## 1 Introduction

Diffusion MRI (dMRI) provides sensitivity to microstructural tissue features in the brain. However, the interpretation of micrometre-scale features from a signal decay measured in a millimetre-scale MRI voxel is at best challenging, and often ill-posed. To address this, biophysical dMRI models aim to separate measured signals into biologically-meaningful parameters, thereby facilitating investigation into the microstructural underpinnings of dMRI data. These models typically describe the tissue as being composed of multiple compartments. For example, in the brain, white matter models typically aim to describe the intra-axonal space, the extra-axonal space, or cellular soma. The parameters associated with each compartment are intended to relate to biologically meaningful characteristics (e.g., soma radius), and are typically estimated by fitting the model to the dMRI signal.

When modelling microstructure in the long diffusion time regime, the brain white matter (WM) is typically described with the “standard model” (Novikov et al., 2018) as follows. The intra-axonal compartment is modelled as impermeable cylinders of negligible radii (“sticks”) to represent the restricted diffusion of water molecules within axons. The extra-axonal compartment uses an axially symmetric diffusion tensor to capture hindered diffusion in the extracellular space. Both intra- and extra-axonal compartments are typically convolved with a fibre orientation distribution function to model fibre dispersion. If cerebrospinal fluid (CSF) is present, an unrestricted isotropic compartment (“ball”) may also be added, leading to a total of nine free parameters. The WM standard model can be extended to describe other tissue features (e.g., a sphere compartment being used to describe signal contributions from cell soma), though this requires the estimation of more parameters.

Fitting even the standard model to “typical” dMRI data (long diffusion time, multi-shell data with b<4 $ms/\mu m^{2}$) is very challenging, if not impossible, as the parameter-fitting landscape is often flat (Jelescu et al., 2020; Novikov et al., 2019). Hence, the standard model is degenerate, with multiple parameter sets fitting the signal equally well. One approach to address this issue is to acquire more comprehensive data with an acquisition specifically designed to incorporate useful new information that stabilises the fitting. For example, using multiple diffusion encodings has been shown to provide robust estimates of the standard model parameters (Coelho et al., 2019; Lampinen et al., 2020; Reisert et al., 2019). For dMRI data that have been acquired using a more “typical” linear diffusion encoding protocol with high angular resolution and/or multiple shells, only relatively few (<5) biophysical parameters can be reliably fitted (Jelescu et al., 2016). Here, a common approach is to constrain parameters such that a restricted biophysical model can be fitted to the dMRI data reliably. Neurite orientation dispersion and density imaging (NODDI) is a constrained form of the standard model, where diffusivities are related and/or fixed to pre-defined values (Zhang et al., 2012). By adopting these assumptions, NODDI only requires the estimation of five free parameters, facilitating more robust parameter estimation. These fitted model parameters can then be compared between two conditions (e.g., patients vs. controls). However, if the model assumptions are inaccurate or a bad representation of the tissue, the resulting parameter estimates may be biased (Howard et al., 2022; Jelescu et al., 2016; Lampinen et al., 2019; Reisert et al., 2017) and the groupwise (patient/controls) comparisons misleading. The fact that NODDI fixes the diffusivities can be challenging for both in-vivo and postmortem studies, where diffusivities in the latter are largely unknown and biased parameter estimates may lead to spurious relationships when correlated with microscopy.

The Bayesian EstimatioN of CHange (BENCH) framework aims to identify the biological underpinnings of the observed signal changes without having to undertake the highly problematic step of fitting the complete model to the data (Rafipoor et al., 2022). BENCH uses a generative model to simulate how the dMRI signal would change if a single model parameter, or set of parameters, was different between the conditions. That is, BENCH defines “change models” in which the change in one model parameter can then be compared with variability in dMRI signals from real data to infer which model parameter best explains the observed change in signal. Crucially, the tissue model does not need to be inverted to infer which parameters are consistent with the observed change, facilitating the use of otherwise degenerate biophysical models that cannot be explicitly inverted. However, a current limitation of BENCH is that it can infer changes in only a single model parameter or a pair of parameters (e.g., $f_{ex}-f_{in}$ , where an increased extra-axonal compartment replaces the intra-axonal compartment).

In this study, we use BENCH to ask whether the dMRI signal measured from the WM possesses sensitivity to the underlying variability in axons and/or cell bodies. Cell bodies or soma are often excluded from data analysis due to increased model degeneracy when including a soma compartment, as it is challenging to distinguish between restricted and unrestricted isotropic compartments. Nevertheless, the density of soma in WM—attributed predominantly to glia—has relevant clinical implications, particularly with respect to neuroinflammation (Jha et al., 2012). BENCH provides a framework in which you can identify which model parameters would be implicated in a given biophysical parameter change, where the latter are explicitly derived from microscopy data in the same tissue as dMRI data. In traditional validation studies, a constrained model would be first fitted to the dMRI data voxel-wise, and the fitted parameter estimates correlated with microscopy-derived metrics acquired in the same tissue sample. Here, parameter degeneracy leads to two issues. First, certain compartments—such as a sphere representing glia soma—would often be excluded from the biophysical model to facilitate fitting. Second, model constraints during fitting can again lead to biased parameter estimates that may affect comparisons with the microscopy.

Circumventing these issues, we developed “continuous BENCH” to relate changes in dMRI signal to variations in the density and dispersion of axons or cell soma extracted from microscopy. This new framework is termed continuous BENCH because it extends the existing BENCH framework to infer changes associated with continuous variables (microscopy-derived metrics), rather than categorical differences (patients vs. controls). Continuous BENCH is first tested on synthetic data generated using numerical simulation of microstructure mesh substrates, providing proof-in-principle of detecting axon and soma density. Continuous BENCH was then applied to an ex-vivo macaque brain with co-registered dMRI and microscopy data. The specific microscopy stains target axons and cell soma, enabling the derivation of metrics of dispersion and density. These metrics (termed “continuous variables”) provided the axis along which we characterise the direction of change in our measured dMRI signal. We then used continuous BENCH to estimate which biophysical model parameter best explains the measured change in the dMRI signal due to microscopy-derived differences in axons and glia.

## 2 Methods

### 2.1 Continuous BENCH

**Fig. 1.**
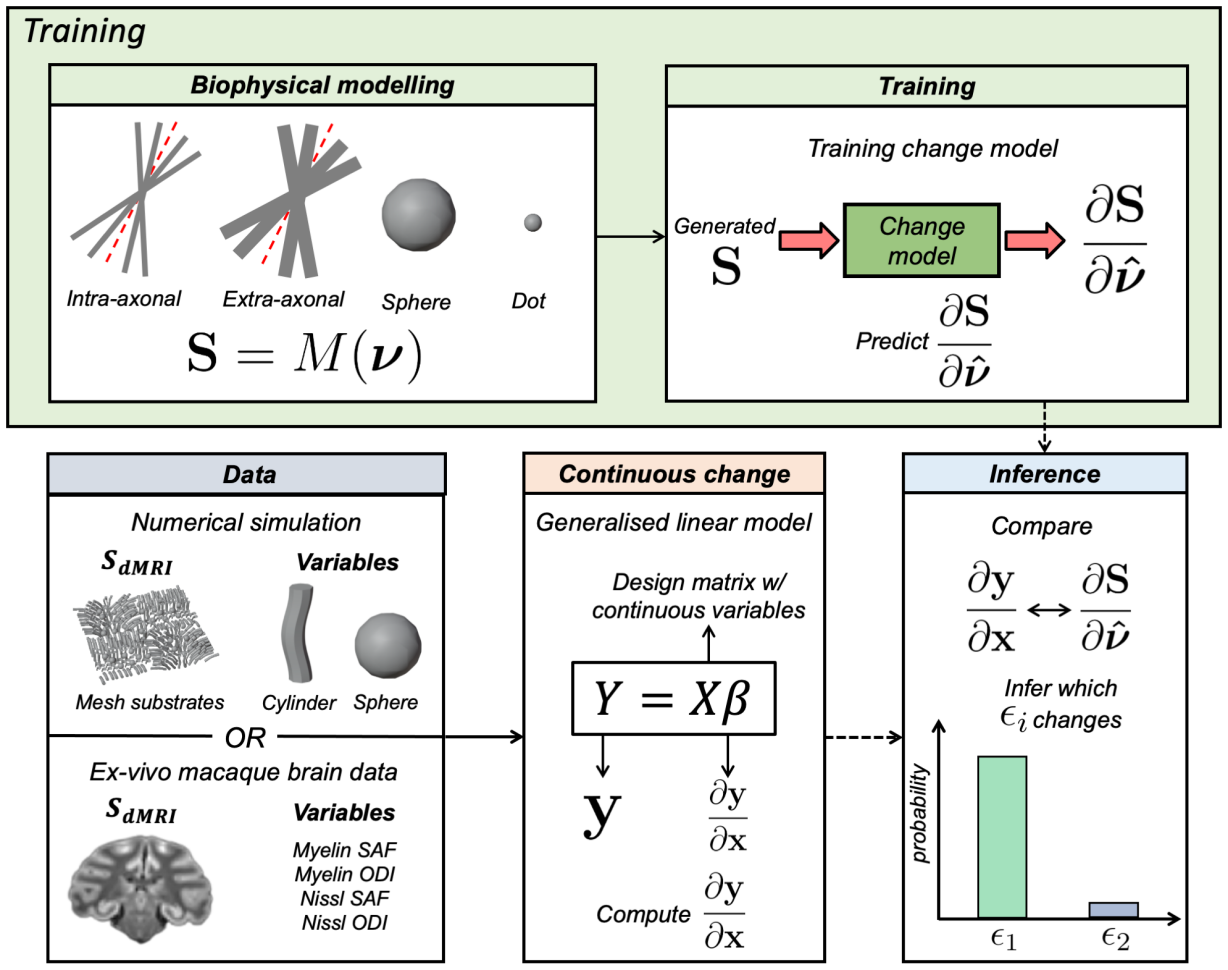
An overview of continuous BENCH. First, in BENCH’s training stage (green box; “Training”), we aim to infer changes in the generated dMRI signal with respect to changes in biophysical parameters $\left(\frac{\partial S}{\partial \hat{v}}\right)$. This is achieved by training “change models” (i.e., regression models) for each biophysical parameter ($\epsilon_{i}$) from a designated diffusion biophysical model (M) using generated data. Next, we quantify how the measured dMRI signal changes due to our continuous variables-of-interest $\left(\frac{\partial y}{\partial x}\right)$ using a generalised linear model (GLM) (beige box; “Continuous change”). In this study, we separately consider numerically simulated and real ex-vivo macaque brain data (blue box; “Data”). The simulated data are to demonstrate continuous BENCH under conditions where the ground truth is known, while the ex-vivo brain data show continuous BENCH applied to real data. Finally, the inference stage (blue box; “Inference”) uses Bayes’ rule to calculate the probability of a given biophysical parameter driving the measured signal variability.

The continuous BENCH pipeline consists of three stages (Fig. 1): the training stage (green box; “Training”), setting up a generalised linear model (GLM) (beige box; “Continuous change”), and the inference stage (blue box; “Inference”). Note that vectors are given in bold. The hat notation is used to represent a unit change in the given vector (i.e., a direction).

First, BENCH’s training stage (Section 2.1.2) uses a generative model (M) to train “change models” where, for a set of input biophysical parameters ($v=[\epsilon_{1},\;\epsilon_{2},\;\ldots \;]$ , where $\epsilon_{1}$ and $\epsilon_{2}$ denote different biophysical parameters), we generate a dMRI signal (S) and a series of “change vectors” $\left(\frac{\partial S}{\partial \hat{v}}\right)$ which describe the change in the dMRI signal induced by a unit change ($\hat{v}$ ) in the biophysical model parameters (v). Next, in real data (y), we use a generalised linear model (GLM) (Section 2.1.3) to compute how the observed dMRI signal changes with respect to some continuous variable(s) of interest (x). In our case, x is the axon or cell body density derived from microscopy, or both. The output of the GLM $\beta =\frac{\partial y}{\partial x}$ defines the “direction of change” of interest. Finally, BENCH’s inference stage (Section 2.1.4) compares the observed $\frac{\partial y}{\partial x}$ with the predicted outputs from change models $\frac{\partial S}{\partial \hat{v}}$ to infer which biophysical parameter (if any) best explains the observed differences in our dMRI signal due to the continuous variables-of-interest. The quality of fit, measured by the chi-squared distance between predicted and observed changes, determines how well the biophysical parameter set $\hat{v}$ explains the dMRI signal differences.

The crux of BENCH is that by reframing the question to consider the MRI signal change across conditions (across subjects, timepoints or voxels), rather than fitting the model to each datapoint separately, we can circumvent inverting the model. This enables investigation of otherwise intractable models. BENCH (i) assumes that, regardless of the absolute parameter values, a change in a given set of parameters alters the signal in a consistent direction, and (ii) aims to identify candidate parameters consistent with the observed change. If multiple parameters could be responsible, BENCH can only make a statement about the probability of each.

#### 2.1.1 Theory

Given a biophysical model (M) that predicts a dMRI signal (S) with input biophysical parameters (ν), S=M(ν), we aim to identify one set of biophysical parameters whose change ($\hat{\nu }$ ) best predicts a measured change in the dMRI signal (Δy ) relative to a baseline dMRI measurement y. Note that S represents the model-predicted dMRI signal, while y is the measured dMRI signal. S and y contain rotationally invariant “summary measures” of the dMRI signal, rather than the raw signal itself. Here, we use the rotationally invariant l=0 and l=2 dMRI signals from two b-value shells, fitted using spherical harmonics where l is the spherical harmonic order. The l=0 term represents the mean signal, while l=2 summarises signal anisotropy.

**Fig. 2.**
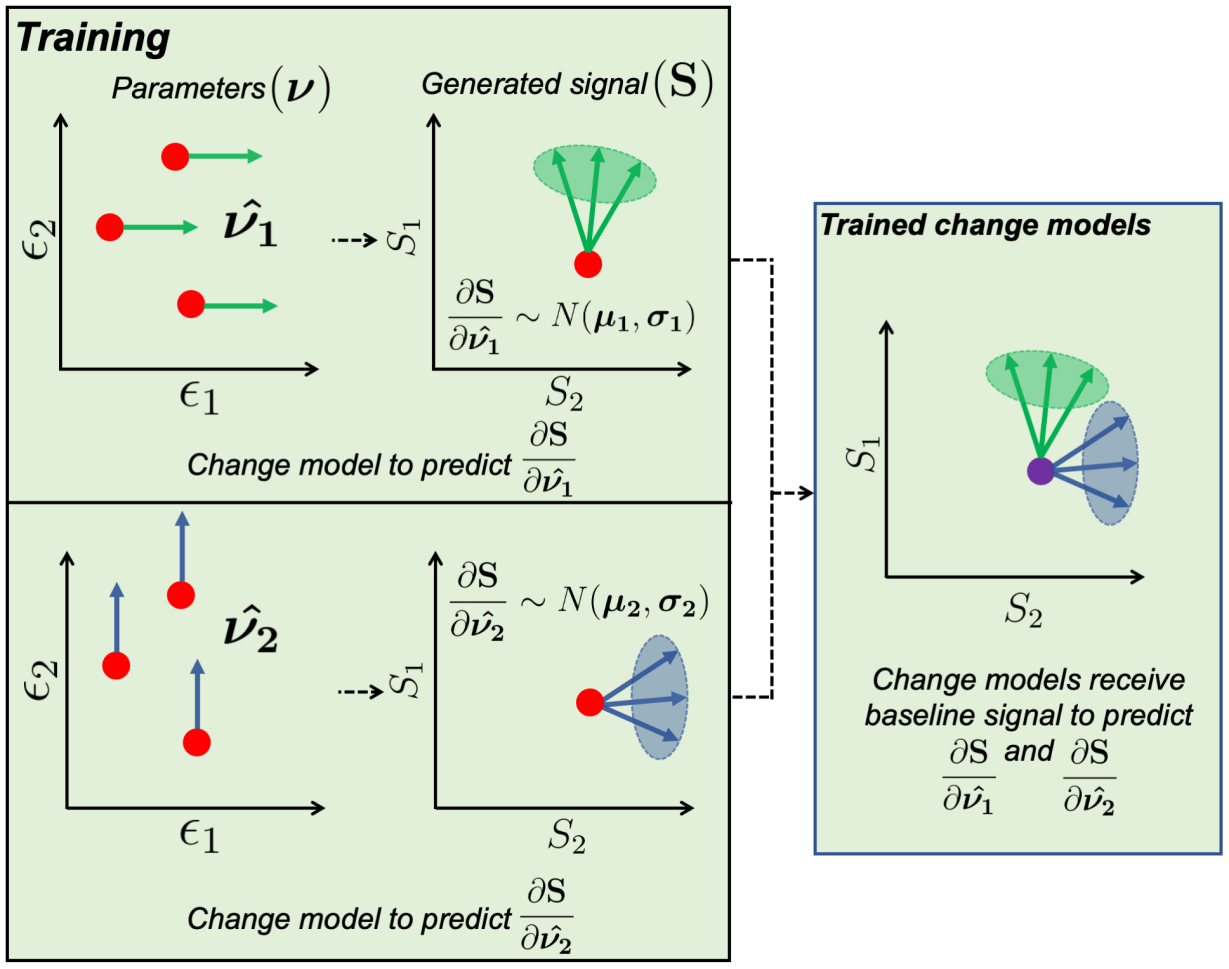
Illustration of the training stage. This is done in the context of a degenerate diffusion biophysical model with biophysical parameters $\epsilon_{1}$ and $\epsilon_{2}$. As the model is degenerate, several parameter combinations (red points, parameters graph) generate the same signal (red point, generated signal graph). During the training phase (box titled “Training”), we aim to train a separate change model for each parameter. For example, while training the model for $\epsilon_{1}$, we calculate the signal for a given pair of $\epsilon_{1}$ and $\epsilon_{2}$ and the signal change due to a small perturbation in $\epsilon_{1}$ only ($\hat{\nu_{ı}}$, a green arrow in the parameters graph) to produce a change vector in the signal space ($\frac{\partial S}{\partial \hat{\nu_{ı}}}$, a green arrow in the generated signal graph). If we repeat this for many combinations of $\epsilon_{1}$ and $\epsilon_{2}$, we can estimate a normal distribution of change vectors (green ellipse $N(\mu_{1},\sigma_{1})$ ). This process is repeated for all parameters in our biophysical model (e.g., $\epsilon_{2}$) to generate their corresponding change models. These trained change models (box titled “Trained change models”) can, thus, provide robust estimates of which parameter has changed (if the parameter distributions/Gaussians are distinct and point in different directions) even when the baseline signal (purple point) itself is degenerate.

In BENCH (Rafipoor et al., 2022), we bypass model inversion by instead inferring $P(\hat{\nu }|\text{y},\;\Delta \text{y})$ , the probability of a change in a given set of biophysical parameters ($\hat{\nu }$ ) given a baseline dMRI signal (y) and a measured change in dMRI signal (Δy ). Using Bayes’ rule (see Supplementary Materials for details), this can be approximated by the likelihood P(Δy|y,ν) , which can be achieved by first computing $P(\Delta \text{y}|\text{y},\hat{v},\;\;\left|\Delta \nu \right|)$ , taking account for both the direction $\hat{\nu }$ and the magnitude |Δν| of the change in the parameter set, and then marginalising with respect to |Δν|. We assume $P(\Delta y|y,\hat{v},\;\left|\Delta \nu \right|)$ can be described by a normal distribution that characterises the spread of possible change vectors originating from some baseline signal y (Fig. 2):

$$P(\Delta y|y,\hat{\nu },\;\left|\Delta \nu \right|)=N(\left|\Delta \nu \;\right|\mu \left(y\right),\;|\Delta \nu {|}^{2}\sigma \left(y\right)+\sigma_{l}),$$ (1)

We estimate μ(y) and σ(y) using trained change models, as outlined below (Section 2.1.2). $\sigma_{l}$ is the noise covariance matrix of the continuous variable (indexed l) as estimated from the data (Section 2.3).

#### 2.1.2 Training change models

As described above, BENCH consists of three stages: training, GLM, and inference. The objective of the first (training) stage is to estimate a distribution of change vectors N(μ,σ) for each biophysical parameter. Figure 2 outlines how this is done in the context of an example degenerate biophysical diffusion model that has input parameters $\epsilon_{1}$ and $\epsilon_{2}$ (i.e., $\nu =[\epsilon_{1},\;\epsilon_{2}]$ ) and outputs the dMRI signal(s) $S=[S_{1},\;S_{2}]$ . For example, $\epsilon_{1}$ and $\epsilon_{2}$ could represent diffusivity and dispersion, while S represents the powder average dMRI signal for two different b-values. Because this is a degenerate model, multiple parameter sets (combinations of $\epsilon_{1}$ and $\epsilon_{2}$) may lead to the same generated dMRI signal S.

In practice, a separate training dataset is generated for each change model (indexed i, where i=1, … , N ). N is the total number of change models considered. Typically, N corresponds to the number of parameters in the model, but this may not always be the case. For example, N can be larger if the models capture interactions where one parameter increases as another decreases.

In this study, we trained individual change models that either characterise a change in a single biophysical parameter or a change in two parameters. The latter case applies only to signal fractions, as the signal fractions are normalised to sum to one. Thus, a change in one compartment’s signal fraction necessitates a complementary change in another compartment’s signal fraction, such as ($f_{in}-f_{ex}$ ), ($f_{in}-f_{sph}$ ), or ($f_{ex}-f_{sph}$ ).

For change model i, the training dataset is generated using the following steps:

1. Sample a parameter combination ($\nu =[\epsilon_{1},\;\epsilon_{2},\;\ldots \;]$ ) from a set of prior distributions. These distributions are predefined empirically.
2. Generate a baseline dMRI signal (S=M(ν) )
3. Induce a small change in the chosen parameter ($\Delta \nu_{i}=\left|\Delta \nu_{i}\right|\hat{\nu_{i}}$). The magnitude of change is drawn from a uniform distribution. Here, $\Delta \nu_{i}\;$ represents a change in the parameter combination required as training data to train change model i.
4. Generate a new dMRI signal ($S=M(\nu +\Delta \nu_{i})$ ) with the new parameter combination.
5. Compute the corresponding change vector $\frac{\partial S}{\partial \hat{\nu_{i}}}$, given $\Delta \nu_{i}$, using:
$$\frac{\partial S}{\partial \hat{\nu_{ı}}}\approx \frac{M(\nu +\Delta \nu_{i})-M\left(\nu \right)}{\left|\Delta \nu_{i}\right|},$$ (2)
  where we assumed that dMRI signals change linearly with the parameter.
6. Repeat steps 1 to 5 to generate a predefined number of pairs of $\left(\frac{\partial S}{\partial \hat{\nu_{ı}}},\;S_{a}\right)$.

These steps are repeated for all parameters of the biophysical model M.

Up to this point, we have estimated $\frac{\partial S}{\partial \hat{\nu_{ı}}}$ for a discrete set of generated S. To describe the distribution of change vectors $N(\mu_{i},\sigma_{i})$ for any S, we assume that $\mu_{i}$ and $\sigma_{i}$ will vary smoothly over signal space such that $\mu_{i}\left(S\right)$ and $\sigma_{i}\left(S\right)$ can be estimated using regression models trained on our generated pairs $\left(\frac{\partial S}{\partial \hat{\nu_{ı}}},S\right)$, details of which are provided in the Supplementary Materials.

#### 2.1.3 Extending BENCH to continuous variables

Next, we use real data to define the baseline dMRI signal y and signal change Δy that are meaningful to our research question (y is used to describe the observed diffusion signal, while S is the generated signal from the forward model). This allows us to calculate the signal change $\left(\frac{\partial y}{\partial x}\right)$ with respect to variable(s)-of-interest x.

Previously, Rafipoor et al. defined x to be a categorical variable describing two discrete groups (healthy controls and patients) (Rafipoor et al., 2022). Specifically, x was a vector where healthy controls were denoted as 0 and patients represented by 1. However, many research problems cannot be formulated in terms of categorical variables of this kind.

We extend the BENCH framework to consider continuous variables (here, histological stains that may have relevance to dMRI signals). We relate changes in the dMRI signal to continuous variables-of-interest via a general linear model (GLM), in the case where there are q continuous variables:

$$Y_{n\;\times \;m}=X_{n\;\times \;\left(q+1\right)}\beta_{\left(q+1\right)\;\times \;m},$$ (3)

where:

$$\left[\begin{array}{cccc} y_{1,1} & y_{1,k} & \cdots & y_{1,m}\\ y_{j,1} & y_{j,k} & \cdots & y_{j,m}\\ \vdots & \vdots & \ddots & \vdots \\ y_{n,1} & y_{n,k} & \cdots & y_{n,m} \end{array}\right]=\left[\begin{array}{ccccc} 1 & x_{1,1} & x_{1,l} & \cdots & x_{1,q}\\ 1 & x_{j,1} & x_{j,l} & \cdots & x_{j,q}\\ \vdots & \vdots & \vdots & \ddots & \vdots \\ 1 & x_{n,1} & x_{n,l} & \cdots & x_{n,q} \end{array}\right]\left[\begin{array}{cccc} \beta_{mean,1} & \beta_{mean,k} & \cdots & \beta_{mean,m}\\ \beta_{1,1} & \beta_{1,k} & \cdots & \beta_{1,m}\\ \vdots & \vdots & \ddots & \vdots \\ \beta_{q,1} & \beta_{q,k} & \cdots & \beta_{q,m} \end{array}\right].$$ (4)

Here, j=1, …, n are the different measurements (e.g., subjects or voxels) for which we have k=1, …, m dMRI summary measures and *l=1, …, q* continuous variables. The dMRI summary measures are rotationally invariant and derived from spherical harmonics fitted to the raw dMRI signal (see Section 2.2.1 for more details). As with any GLM, the βs are estimated by multiplying the matrix of summary measures, Y, with the pseudoinverse of the design matrix, X.

#### 2.1.4 Inference

**Fig. 3.**
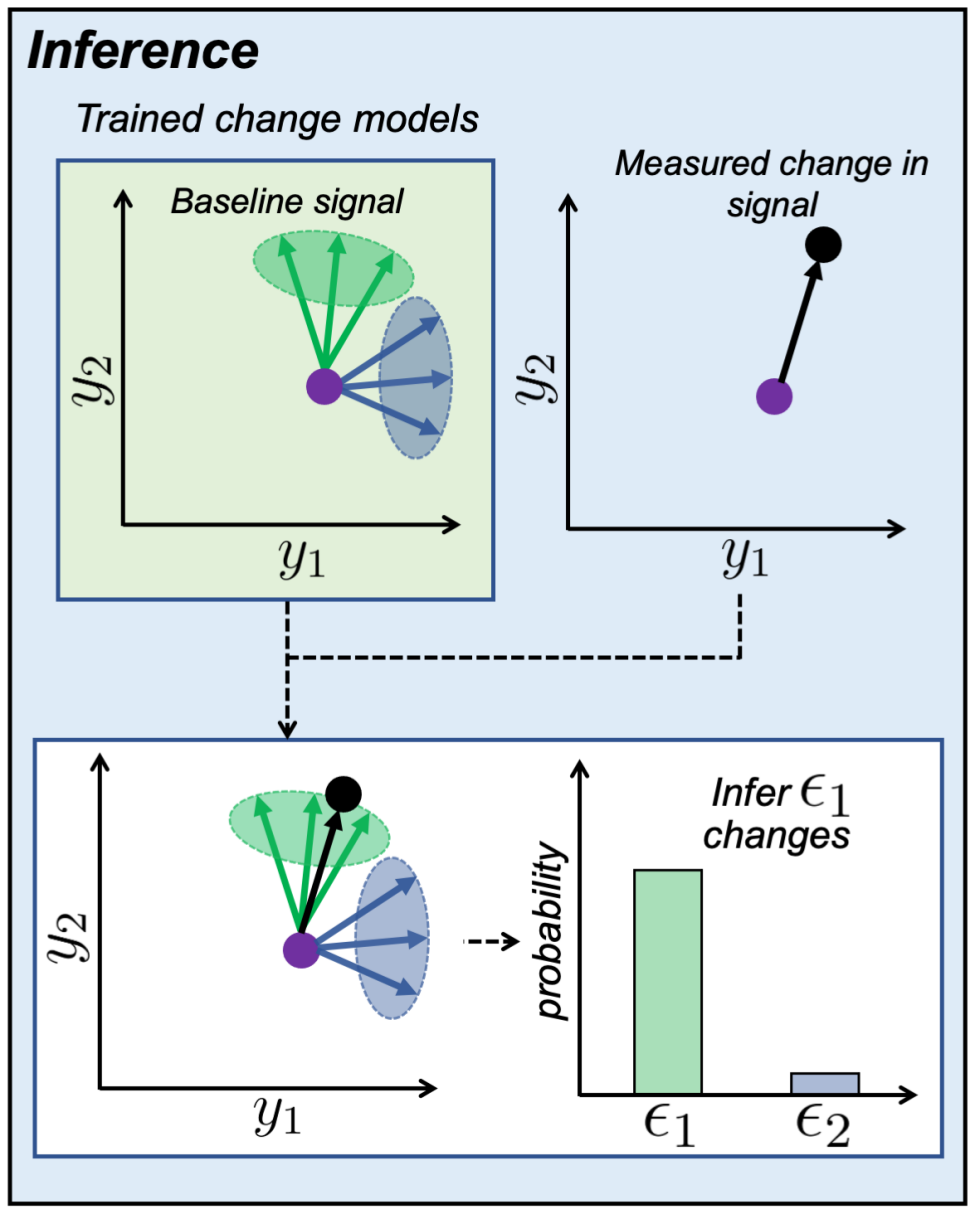
Illustration of the inference stage. With the trained change models and a baseline dMRI signal (purple dot), we can generate a distribution of change vectors for $\epsilon_{1}$ (green ellipse) and $\epsilon_{2}$ (blue ellipse). We then compare a measured dMRI signal change associated with a continuous variable (purple to black dot) with the distribution of change vectors. This allows us to estimate the probability of each biophysical parameter explaining the measured change in the dMRI signal (Equation 1 in Supplementary Materials). In this case, we would infer that the observed dMRI signal change is mostly likely due to a change in $\epsilon_{1}$.

The inference stage (Fig. 3) combines outputs from the training stage $\frac{\partial S}{\partial \hat{\nu_{ı}}}$ and the GLM $\frac{\partial y}{\partial x}$ to infer which change model best explains the observed change in dMRI with respect to the continuous variables-of-interest.

The first row of the GLM, $\beta_{mean}=[\beta_{mean,1},\;\ldots ,\;\beta_{mean,m}]$ , is our data-driven estimate of the baseline dMRI signal. $\beta_{mean}$ is inputted into the trained change models to determine $N(\mu_{i}(\beta_{mean}),\sigma_{i}(\beta_{mean}))$ for each biophysical parameter.

Next, we compute the measured change in dMRI signal (relative to $\beta_{mean}$ ) due to each continuous variable $x_{l}$. Instead of evaluating all variables at once, we assess how each one individually influences dMRI signals while holding others constant, isolating its contribution to the observed changes in dMRI signals. This measured change is computed by multiplying the variable’s direction of change $\left(\frac{\partial y}{\partial x}=\beta_{l}\right)$ with its change effect size ($\delta_{l}$), where $\delta_{l}$ is measured directly from the data. To account for measurement noise in the dMRI signal, a noise covariance matrix ($\sigma_{l}$ in Equation 1) is also estimated directly from the data. The estimation of $\delta_{l}$ and $\sigma_{l}$ is described later in Section 2.3.

Putting this together (Equation 1), for each continuous variable l and change model i, we evaluate the likelihood P(Δy=βlδl |  y=βmean, νl^) . Using this likelihood in Bayes’ rule (Equation 1 in Supplementary Materials) gives the probability of a change in biophysical parameter $\epsilon_{i}$ explaining our data, $P(\hat{\nu_{ı}}\;|y=\beta_{mean},\;\Delta y=\beta_{l}\delta_{l})$ . These likelihoods are normalised across all biophysical parameters and inference is performed separately for each continuous variable-of-interest.

### 2.2 Data simulation and acquisition

#### 2.2.1 Numerical simulations

**Fig. 4.**
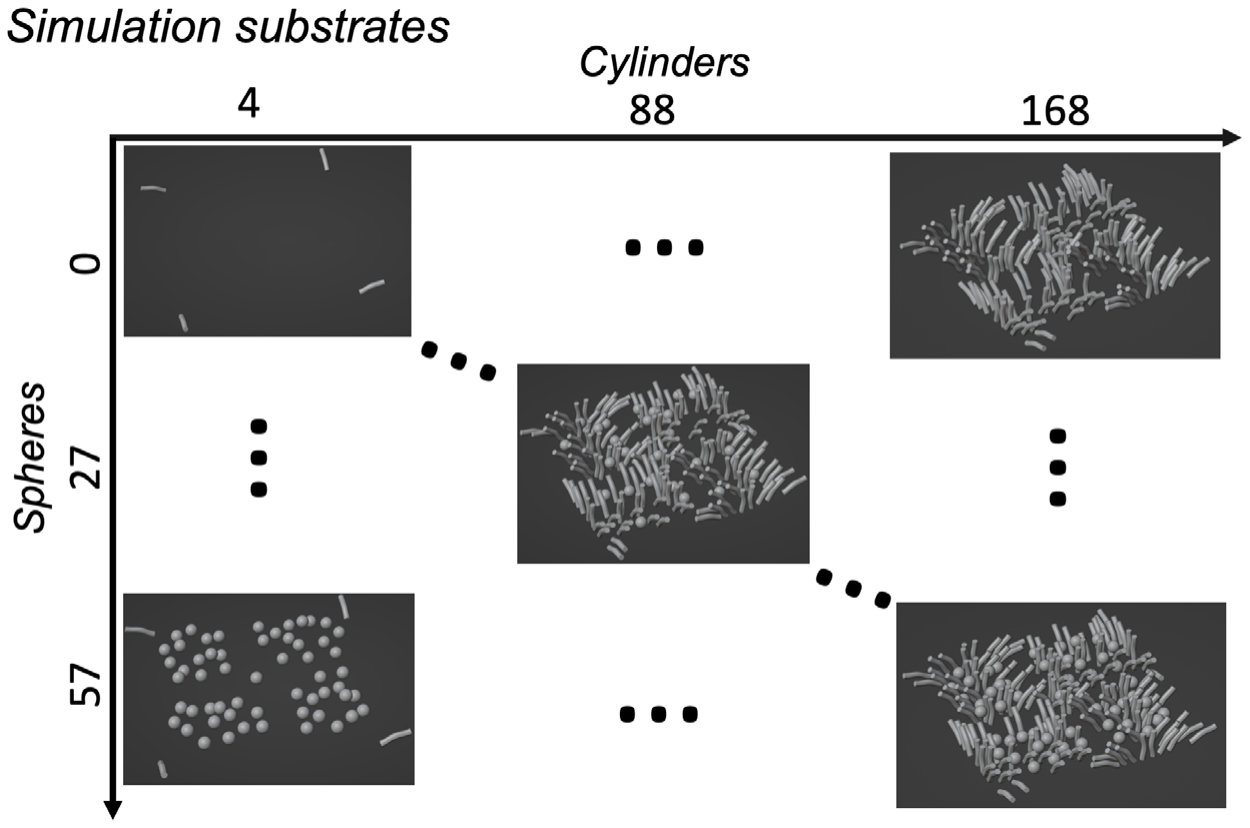
The mesh substrates used for simulation. A total of 41 substrates were used, each with a different number of cylinders (representing myelinated axons; radius = 2.5 μm ; Veraart et al., 2020) and spheres (representing glial cell soma; radius = 5 μm ; Howard, Huszar, Zhu, et al., 2023).

Monte Carlo simulations (Cook et al., 2006) of spins diffusing in a geometric space designed to mimic the WM were performed to establish whether our method can detect subtle changes approximating the density of axons and soma. A total of 41 artificial 3D meshes (Fig. 4) were created with axons as undulating cylinders (radius = 2.5 μm (Veraart et al., 2020); undulation amplitude = 1 μm ) and cellular soma as spheres (radius = 5 μm , which is within the observed range of glia cell soma size; Howard, Huszar, Zhu, et al., 2023). The cylinder radius of 2.5 μm was chosen to align with the dMRI effective axon radius, which accounts for the enhanced dMRI sensitivity to larger diameter axons (Veraart et al., 2020). Axons were oriented in each substrate to simulate fibre dispersion, approximately following a Watson distribution (Zhang et al., 2012). Substrates (size = 200 ×  200 × 25 $\mu m^{3}$) were generated for various numbers of undulating cylinders (n = [4, 48, 88, 168]) and spheres (n = [0, 9, 15, 21, 27, 33, 39, 45, 51, 57]), which resulted in substrates with varying intra-axonal (0 to 8.25% of substrate) and sphere (or intra-soma) volume fractions (0 to 3% of substrate, in line with microglia cell density we previously observed in postmortem human brain WM; Kor et al., 2022). Simulations were performed using 160,000 spins (uniformly initialised in both intra- and extra-axonal space) with a time step of 8.62 μs (Hall & Alexander, 2009) and impermeable membranes. To simulate ex-vivo tissue conditions, bulk water diffusivity was set to 1 $\mu m^{2}/ms$ . dMRI signals were simulated for each substrate using acquisition parameters very similar to what was previously used to acquire ex-vivo macaque brain HARDI data in Howard, Huszar, Smart, et al. (2023), except with a reduced number of gradient directions: 90 gradient directions acquired each at b = 7 and 10 *$ms/\mu m^{2}$* and 18 images acquired at b = 0 $ms/\mu m^{2}$. Other parameters include: Δ=  24 ms , TE= 42.5 ms , G= 15.9, 19.1 G​/​cm , δ= 14 ms .

BENCH is designed to work with rotationally invariant summary measures of the signal, rather than the raw dMRI signals themselves. To achieve this, spherical harmonics (Kazhdan et al., 2003; Novikov et al., 2018; Tournier et al., 2004) were fitted to the dMRI signal and the summary measures were calculated as the weighted average of the squared coefficients for degree ℓ= 0 and 2. This resulted in 4 rotationally invariant summary measures (2 summary measures × 2 shells) per mesh substrate. For more details, please refer to the Supplementary Materials.

#### 2.2.2 Ex-vivo macaque brain data

We applied continuous BENCH to previously published ex-vivo macaque brain data (Howard, Huszar, Smart, et al., 2023), where postmortem dMRI and co-registered (Huszar et al., 2023) microscopy metrics specific to cell soma and myelin were available. The macaque brain exhibited tissue pathology in some regions due to lesions of the orbitofrontal cortex and a likely cerebral bleed found postmortem (Howard, Huszar, Smart, et al., 2023). Since our study does not focus on differentiating healthy from pathological tissue, we use both natural and pathological variations in tissue to drive BENCH’s inference in microstructural changes. Note that we only describe parts of the data that are directly relevant to this work.

##### 2.2.2.1 Diffusion MRI data

**Fig. 5.**
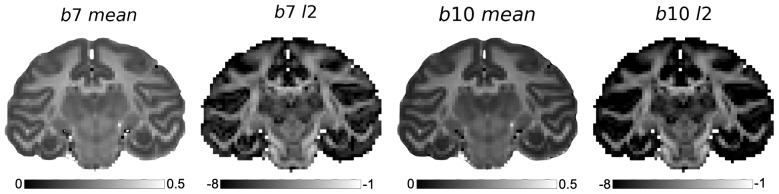
dMRI summary measure maps sampled from the ex-vivo macaque brain data. Analysis was restricted to white matter voxels only. The mean summary measures represent the diffusivity in each shell, averaged across all diffusion directions, while the l2 summary measures estimate the anisotropy, akin to fractional anisotropy (FA).

The acquisition and preprocessing of the ex-vivo HARDI data from a perfusion-fixed brain of an adult rhesus macaque have been previously described in Howard, Huszar, Smart, et al. (2023). Briefly, data were acquired on a 7T small animal scanner (Agilent) with 1,000 gradient directions at b = 7, 10 $ms/\mu m^{2}$, along with 80 images acquired at negligible diffusion-weighting (b = 0 $ms/\mu m^{2}$). Images were acquired with 1 mm isotropic resolution. Other acquisition parameters are identical to what was used for the numerical simulation. A WM mask was generated (Howard, Huszar, Smart, et al., 2023) (Supplementary Materials Fig. S1). Rotationally invariant summary measures were similarly calculated from the dMRI signal. Representative dMRI summary measure maps are provided in Figure 5.

##### 2.2.2.2 Microscopy data

Following MRI data acquisition, the macaque brain was sectioned along the anterior-posterior axis to produce coronal tissue sections. Of relevance to this study, 50 μm thick sections were histologically stained for Cresyl violet to visualise Nissl bodies, and Gallyas silver, which targets myelin (Howard, Huszar, Smart, et al., 2023). Each stain was repeated every 350 μm throughout the brain. Sections were imaged at a resolution of 0.28 μm per pixel. All microscopy slices were co-registered to the MRI volume using TIRL (Huszar et al., 2023).

The histology images were analysed to extract two quantitative metrics: the stained area fraction (SAF) and the orientation dispersion index (ODI). The SAF is a metric for microstructural stain density, while the ODI describes how dispersed the stained microstructure is. The details of how both SAF and ODI are derived are published in Howard, Huszar, Zhu, et al. (2023). In brief, stain segmentation was performed using data-driven thresholds based on a weighted-Otsu method (Yuan et al., 2015), similar to that implemented in Kor et al. (2022). The SAF was then calculated as the number of positive pixels over a local neighbourhood the size of the MRI voxel. Dispersion was quantified by performing structure tensor analysis to estimate the primary fibre orientation per pixel (Budde & Frank, 2012). A Bingham distribution was then fitted to the set of microscopy-derived orientations within the neighbourhood of an MRI voxel to calculate an ODI that is directly comparable with MRI-derived fibre orientation distributions, albeit in 2D.

Continuous BENCH was applied to co-registered dMRI, Nissl-derived, and myelin-derived data from the anterior brain (17,945 voxels in total). A voxel mask is shown in Supplementary Materials Figure S1 (myelin staining in the posterior brain was excluded as it was found to be corrupted). Supplementary Materials Figure S2 displays the voxelwise correlations between each dMRI summary measure and each microscopy-derived metric for the voxels in this mask, highlighting both their dynamic ranges and how each measure varies relative to each metric.

### 2.3 Data analysis

We applied continuous BENCH to both numerical simulation and ex-vivo macaque brain data. For either dataset, we need to specify:

1. a generative biophysical model M used for training change models (c.f. Section 2.1.2)
2. the measured direction of change $\beta_{l}$ (c.f. Section 2.1.3) and the uncertainty on $\beta_{l}$ (i.e., $\sigma_{l}$, the noise covariance matrix for $l^{th}$ continuous variable) (c.f. Section 2.1.4)

#### 2.3.1 Biophysical model

For both datasets, we used an extended WM standard model that included an intra-axonal compartment modelled as dispersed sticks, an extra-axonal compartment characterised as dispersed zeppelins, and an intra-soma compartment modelled as a sphere using the Gaussian phase distribution approximation (Balinov et al., 1993; Neuman, 1974; Stanisz et al., 1997). A dot compartment was also added to account for stationary water, which is often present in ex-vivo data (Alexander et al., 2010; Panagiotaki et al., 2009). All of these compartments were characterised as either anisotropically (zeppelin, sticks) or isotropically (sphere, dot) restricted diffusing spins. When analysing data simulated from mesh substrates, we also included an isotropic Gaussian (ball) compartment to model freely diffusing spins that do not interact with any membrane. In total, our biophysical models require 10 and 12 model parameters for simulated and real ex-vivo data, respectively. All model parameters are defined in Table 1. In both cases, the signal fractions were assumed to sum to 1.

**Table 1.** Parameters of the extended WM standard model.

Parameter	Units	Description	Range
$f_{in}$	-	Signal fraction for intra-axonal compartment	[0, 1]
$f_{ex}$	-	Signal fraction for extra-axonal compartment	[0, 1]
$f_{sph}$	-	Signal fraction for intra-soma compartment	[0, 0.10]
$f_{dot}$	-	Signal fraction for the dot compartment	[0, 0.10]
$f_{ball}$	-	Signal fraction for ball compartment	[0, 0.10]
ODI	-	Orientation dispersion index	[0.01, 0.50]
$D_{a,in}$	$\mu m^{2}/ms$	Axial diffusivity for the intra-axonal compartment	[0.50, 1.5]
$D_{a,ex}$	$\mu m^{2}/ms$	Axial diffusivity for the extra-axonal compartment	[0.50, 1.5]
$D_{r,ex}$	$\mu m^{2}/ms$	Radial diffusivity for the extra-axonal compartment	[0.50, 1.5]
$D_{sph}$	$\mu m^{2}/ms$	Isotropic diffusivity for the intra-soma compartment	[0.50, 1.5]
$D_{ball}$	$\mu m^{2}/ms$	Isotropic diffusivity for the ball compartment	[0.50, 2.5]
$R_{sph}$	μm	Radius of the intra-soma compartment	[0.01, 10]

When analysing simulation data, a ball compartment (parameters shaded in grey) was added to explicitly model spins that do not interact with any membranes (i.e., when modelling simulation data).

#### 2.3.2 Training change models

**Fig. 6.**
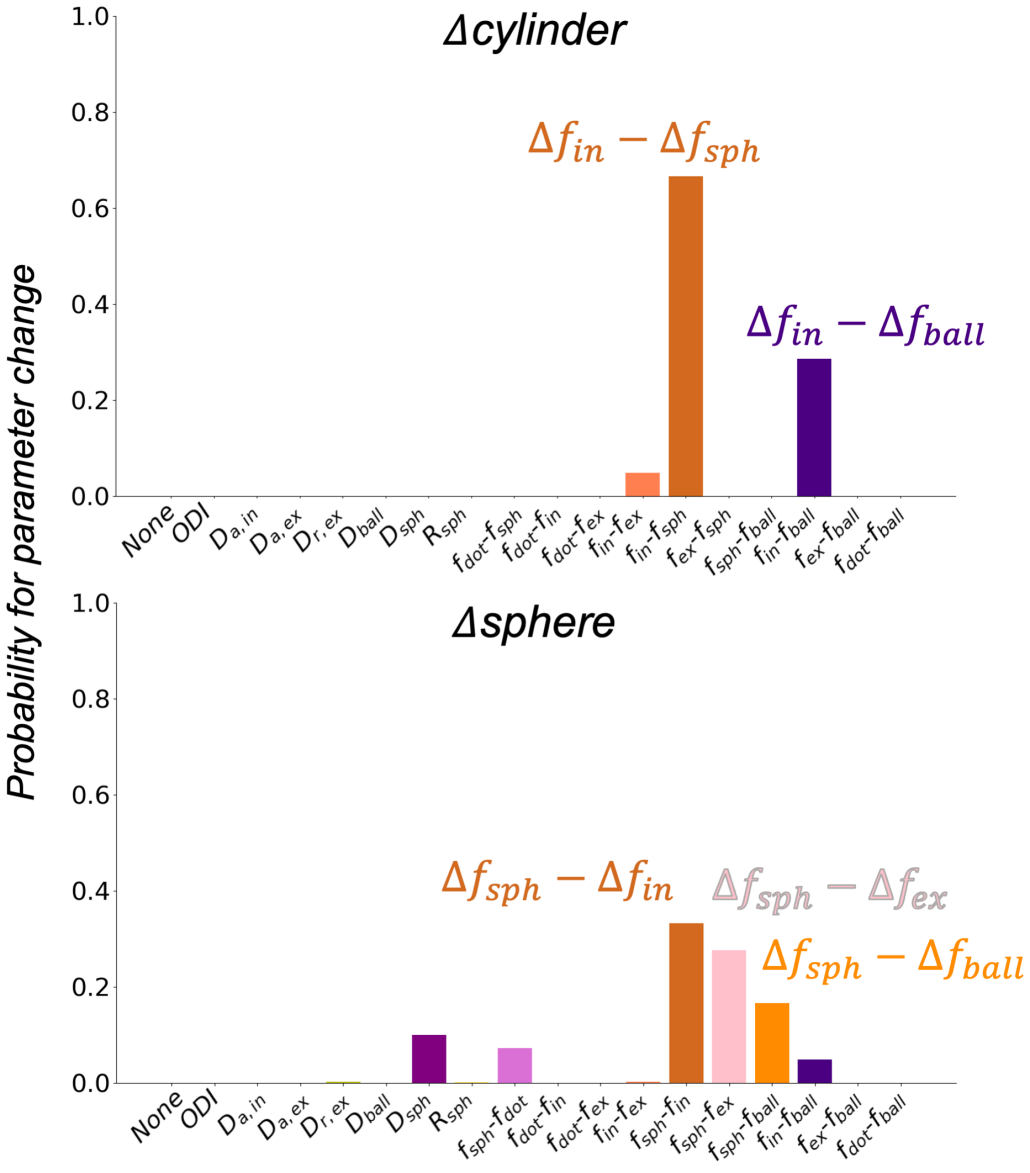
Inference of which biophysical parameter(s) best explains a change in cylinders and spheres in the mesh substrates. The change in cylinders (Δcylinder) was best explained by the intra-axonal signal fraction replacing sphere and ball signal fractions ($\Delta f_{in}-\Delta f_{sph}$ , $\Delta f_{in}-\Delta f_{ball}$ ). The change in spheres (Δsphere) was attributed to an increase in the sphere signal fraction replacing the intra-axonal, extra-axonal, and ball signal fraction ($\Delta f_{sph}-\Delta f_{in}$ , $\Delta f_{sph}-\Delta f_{ex}$ , $\Delta f_{sph}-\Delta f_{ball})$ . Note that for the “Δsphere” plot, the “$\Delta f_{in}-\Delta f_{sph}$ ”, “$\Delta f_{ex}-\Delta f_{sph}$ ” labels on the x-axis were replaced with “$\Delta f_{sph}-\Delta f_{in}$ ”, “$\Delta f_{sph}-\Delta f_{ex}$ ” to reflect that the observed signal change was in the opposite direction.

Each change model was trained using 20,000 pairs of baseline dMRI signal and generated change vector $\left(\text{S},\;\frac{\partial \text{S}}{\partial \hat{\nu_{ı}}}\right)$ produced from the generative biophysical model (c.f. Section 2.1.2). We trained a change model for each biophysical parameter, and combinations of signal fractions (e.g., sphere fraction replacing extra-axonal fraction ($f_{sph}-f_{ex}$ )) such that the signal fractions sum to 1. These change models are referred to and explained in Figure 6 caption.

#### 2.3.3 The GLM

The GLM (c.f. Section 2.1.3) was set up with the microscopy metrics as the explanatory variables. For simulated data, the explanatory variables were the number of spheres and cylinders in each substrate. For the real (ex-vivo) data, the explanatory variables were the four microscopy-derived metrics: the Nissl SAF, Nissl ODI, myelin SAF, and myelin ODI.

The GLM outputs the baseline dMRI signal $\beta_{\text{mean}}$ and the direction of change ($\beta_{l}$) in different metrics, for both the simulated data ($\beta_{\text{cylinders}}$ , $\beta_{\text{spheres}}$ ) and the ex-vivo data ($\beta_{\text{Nissl SAF}}$ , $\beta_{\text{Nissl ODI}}$ , $\beta_{\text{myelin SAF}}$ , $\beta_{\text{myelin ODI}}$ ). For simulated data, $\beta_{\text{mean}}$ is the simulated dMRI signal averaged across all substrates. In ex-vivo, $\beta_{\text{mean}}$ denotes the measured dMRI signal averaged across all voxels in the ex-vivo macaque brain data, encompassing voxels containing both normal and pathological tissue.

For ex-vivo data, we first investigated how each microscopy-derived metric individually explained variance in the dMRI data by performing a separate GLM for each metric (termed “single regressor”). We then determined how each metric uniquely explains variance in the dMRI data by performing multiple linear regression with all metrics in the same GLM design matrix (termed “all regressors”).

As BENCH requires the measured change in the dMRI signal Δy rather than $\beta_{l}$ $\left(where\;\;\beta_{l}=\frac{\partial \text{y}}{\partial \text{x}}\right)$, $\beta_{l}$ was multiplied with the effect size $\delta_{l}$. In the simulated data, $\delta_{l}$ was equal to the range of cylinders (n = 57) and spheres (n = 328), respectively. For the ex-vivo data, the value of $\delta_{l}$ was calculated by taking the difference between the 99.9th percentile and the 0.1th percentile of each microscopy-derived metric’s distribution across the brain.

For the simulated data, the noise covariance $\sigma_{l}$ was taken to be the covariance of $\beta_{l}$ computed from 100 different instances of additive Rician noise (SNR = 150). In the ex-vivo data, the noise covariance was derived from the data via a bootstrapping method (5,000 iterations). In each iteration, we sampled 1,000 voxels from the white matter mask and computed $\beta_{\text{Nissl SAF}}$ , $\beta_{\text{Nissl ODI}}$ , $\beta_{\text{myelin SAF}}$ , and $\beta_{\text{myelin ODI}}$ . $\sigma_{l}$ was taken to be the covariance of $\beta_{l}$ across iterations.

#### 2.3.4 Inference

The measured change in dMRI signal due to different continuous variables ($\beta_{l}\delta_{l}$), along with their noise covariance term ($\sigma_{l}$), were used for inference. In the simulated data, we present the inferred probability averaged across 100 iterations. For ex-vivo data, the final probabilities were generated from a single inference.

## 3 Results

### 3.1 Numerical simulations

Continuous BENCH was applied to simulated data from mesh substrates with differing numbers of cylinders and spheres (Fig. 6) to validate its ability to attribute these changes to appropriate biophysical model parameters (i.e., different $f_{in}$ and $f_{sph}$ ). For simulations with increasing numbers of cylinders, BENCH predicted increasing intra-axonal signal fractions replacing sphere and ball signal fractions, specifically either ($f_{in}-f_{sph}$ ) or ($f_{in}-f_{ball}$ ), with probabilities of ~70% and ~30% respectively. For simulations with increasing numbers of spheres, continuous BENCH mainly inferred changes in the sphere compartment, specifically ($\Delta f_{sph}-\Delta f_{in}$ ), ($\Delta f_{sph}-\Delta f_{ex}$ ) and ($\Delta f_{sph}-\Delta f_{ball})$ , with probabilities ranging from ~20 to 40%.

### 3.2 Ex-vivo macaque brain data

Continuous BENCH was applied to ex-vivo macaque data to relate changes in biophysical model parameters to myelin and Nissl SAF and ODI derived from microscopy. We explored both each metric independently (“single regressor”) and all metrics together (“all regressors”).

#### 3.2.1 Single regressor

**Fig. 7.**
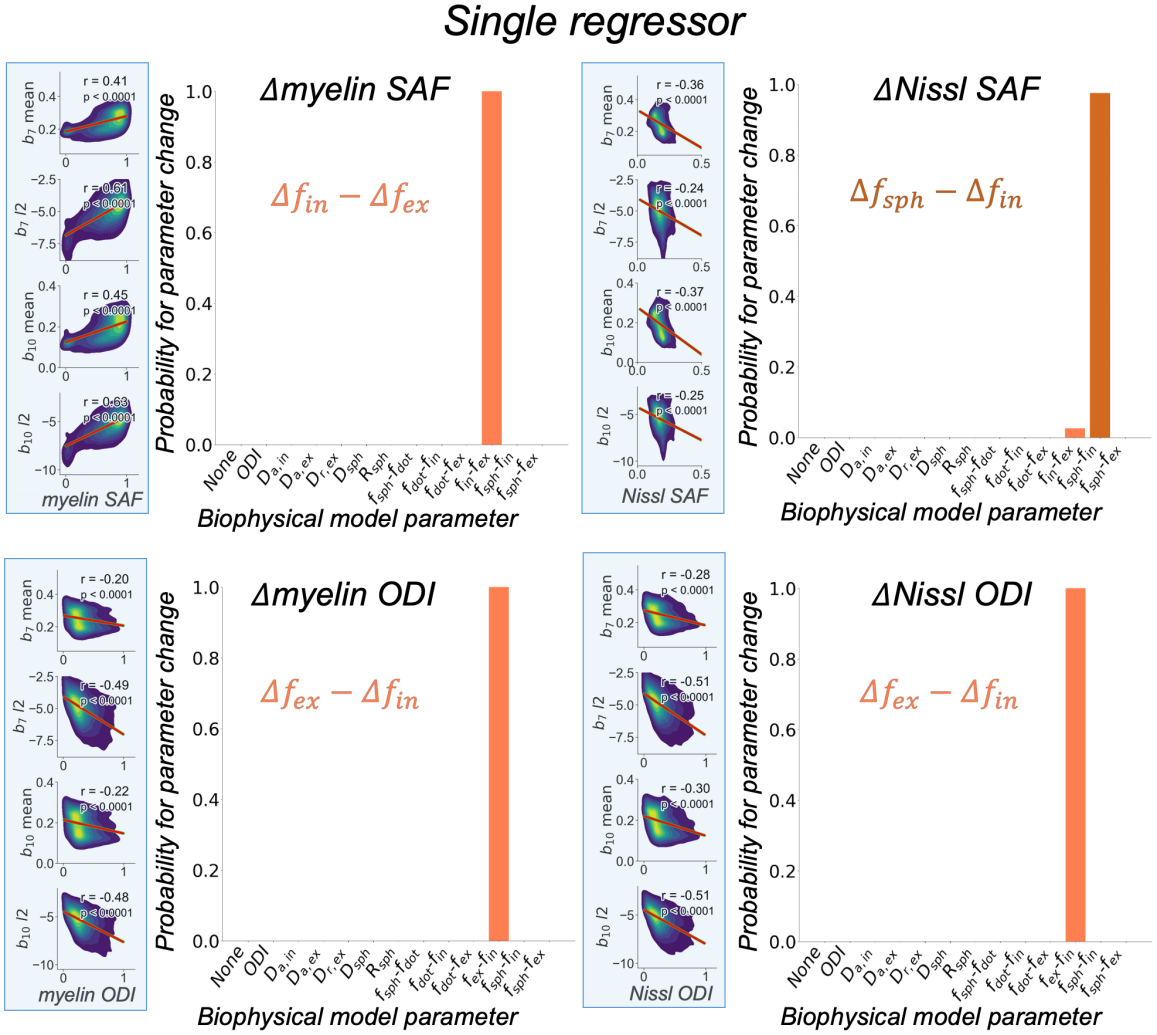
Inferred probabilities on real data when using each microscopy-derived metric (myelin SAF/Nissl SAF/myelin ODI/Nissl ODI) as the only regressor in the GLM. Voxelwise correlations between the dMRI summary measures ($b_{7}$ mean, $b_{7}\;l2$ , $b_{10}$ mean, $b_{10}\;l2$ ) and the microscopy-derived metric are shown on the left hand side of each plot (light blue boxes; see Supplementary Materials Figure S2 for all density plots). The β estimated from the GLM using these data ($\beta_{\text{Nissl SAF}}$ , $\beta_{\text{Nissl ODI}}$ , $\beta_{\text{myelin SAF}}$ , $\beta_{\text{myelin ODI}}$ ) are used by BENCH to infer the probabilities of parameter change shown here (c.f. Section 2.1.4). Based on the inferred probabilities, the change in myelin and Nissl (glia) density (SAF; stained area fraction) was linked to an increase in $f_{in}\;$ and $f_{sph}\;$ respectively. A change in either myelin or Nissl orientation dispersion index (ODI) was attributed to an increase in $f_{ex}$ . In the bottom two plots, the “$\Delta f_{in}-\Delta f_{ex}$ ” label on the x-axis was replaced with “$\Delta f_{ex}-\Delta f_{in}$ ” to reflect that the observed signal change was in the opposite direction.

Figure 7 shows the inference of each parameter in separate GLMs. Note that none of the inferences have been thresholded—the plots display the inferred probabilities as they are. An increase in myelin SAF was linked to an increase in intra-axonal signal fraction replacing the extra-axonal signal fraction ($\Delta f_{in}-\Delta f_{ex}$ ) (probability~100%). The increased density of Nissl was linked to the intra-axonal signal fraction being replaced by the sphere compartment signal fraction ($\Delta f_{sph}-\Delta f_{in}$ ) (probability~100%). An increase in either myelin- or Nissl-derived dispersion (ODI) led to an increase in the extra-axonal signal fraction and a complementary decrease in the intra-axonal signal fraction ($\Delta f_{ex}-\Delta f_{in}$ ) (probability~100%).

#### 3.2.2 All regressors

**Fig. 8.**
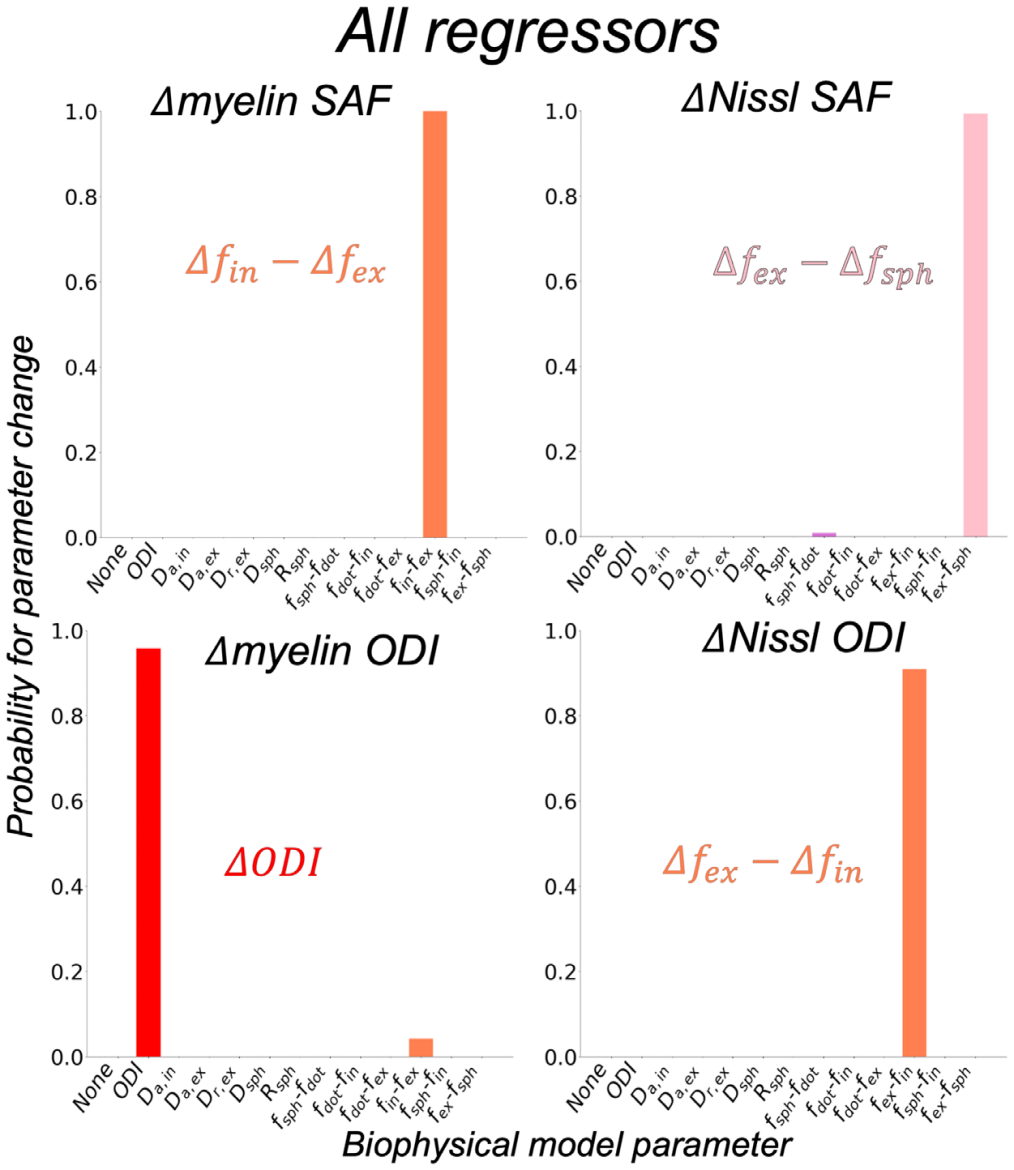
Inferred probabilities when using all microscopy-derived metrics as regressors in the same GLM. Results are shown as similarly described in Figure 7.

When using all metrics simultaneously as regressors in the GLM, the inferred probabilities for myelin-derived ODI and Nissl SAF differed (Fig. 8) from the analysis using each metric independently. Myelin-derived ODI was confidently linked to model parameter ODI (probability~95%), while an increase in Nissl SAF was associated with increased extra-axonal signal fraction replacing the sphere signal fraction ($\Delta f_{ex}-\Delta f_{sph}$ ) (probability~100%).

**Fig. 9.**
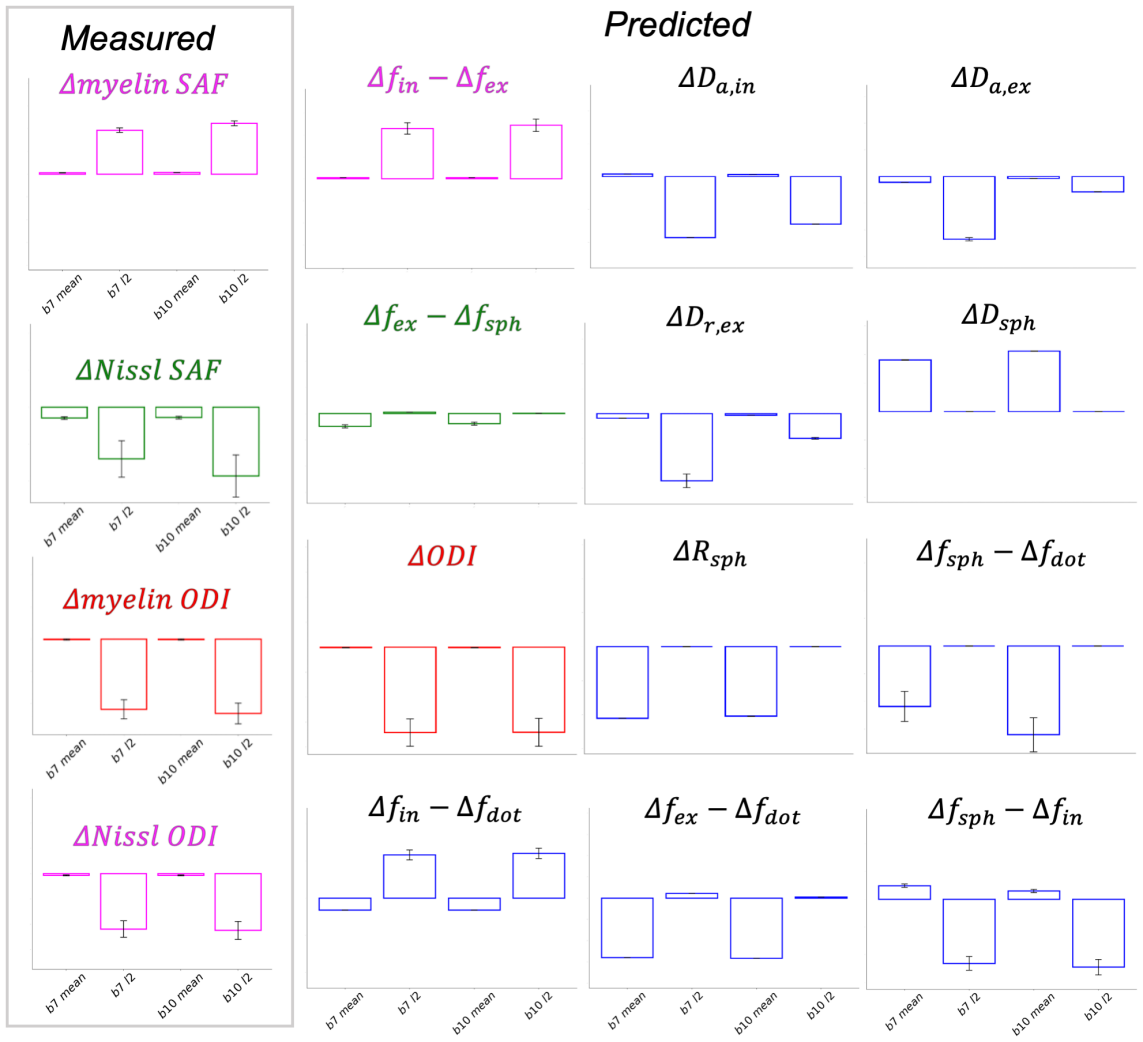
The “pattern of change” in dMRI summary measures (x-axis) for each microscopy metric from the real ex-vivo data (left box) are compared to those predicted from BENCH’s trained change models. In each plot, the dMRI summary measures on the x-axis are the $b_{7}$ mean, the $b_{7}\;l2$ , the $b_{10}$ mean, and the $b_{10}\;l2$ terms. The y-axis describes the change in signal for each summary measure with respect to a unit change in the continuous variable (left “Measured”) or a unit change in the model parameter (right, “Predicted”). The values along the y-axis are omitted as BENCH only considers the relative pattern of change across the summary measures, not the absolute magnitude. The colours (pink, green, and red) show which patterns of change are inferred by BENCH to be the most similar. Note, the myelin SAF is linked to ($\Delta f_{in}-\Delta f_{ex}$ ), while Nissl-derived ODI is linked to the same pattern of change, but in the opposite direction (i.e., $\Delta f_{ex}-\Delta f_{in}$ ).

We further investigated these results by plotting the change in each dMRI summary measure with respect to the microscopy (Fig. 9, left, titled “Measured”). This observed “pattern of change” is compared to BENCH’s trained change models showing the predicted pattern of change for each biophysical parameter (Fig. 9, right, titled “Predicted”). When the measured pattern of change matches one of the predicted patterns of change, BENCH should infer that biophysical parameter with high probability. The chi-squared distance is used to quantify the similarity between the measured and predicted patterns of change (appendix B of Rafipoor et al., 2022). The change model’s pattern of change with the lowest chi-squared distance relative to all other change models is inferred to have the highest probability.

For the myelin SAF and ODI, the measured patterns are highly similar to those from $\Delta f_{in}-\Delta f_{ex}$ and model parameter ODI, respectively. Similarly, Nissl ODI was the most similar to the inverted pattern of change for $\Delta f_{in}-\Delta f_{ex}$ , indicating a change in $\Delta f_{ex}-\Delta f_{in}$ . The patterns of change for myelin SAF and Nissl ODI are very similar but opposite in magnitude, which is why they were matched to $\Delta f_{in}-\Delta f_{ex}$ and $\Delta f_{ex}-\Delta f_{in}$ (the inverse). Though the myelin ODI also looks similar, it differs in that the changes in the $b_{7}$ and $b_{10}$ mean terms (1st and 3rd columns) are effectively zero, whereas the myelin SAF and Nissl ODI show small but non-zero changes in these terms. This difference (i.e., the lack of change in the $b_{7}$ and $b_{10}$ mean terms) results in myelin ODI matching with the ΔODI biophysical parameter. This is as expected as changes in signal fractions, but not dispersion, would affect the $b_{7}$ and $b_{10}$ mean terms (equivalent to the spherically averaged signal), while dispersion affects the *l2* terms only.

The Nissl SAF was more challenging to interpret. The measurable changes in the dMRI signal with respect to the Nissl SAF indicate that dMRI is sensitive to some microstructural feature(s) associated with changes in Nissl SAF, though the measured pattern of change itself did not match any of the predicted patterns of change. The microstructure dMRI is sensitive to probably the cell soma themselves (what we measure in the microscopy) or other correlated features. Here, BENCH assigned $\Delta f_{ex}-\Delta f_{sph}$ as the most similar match, likely due to the similarity of the $b_{7}$ and $b_{10}$ mean. The l2 terms look quite different. As there is high standard deviation in Nissl SAF’s $b_{7}$ and $b_{10}$ l2 summary measures, BENCH will downweight the importance of matching these terms. Ultimately, this result suggests our biophysical model is inadequate at explaining Nissl variation across the brain. This result also highlights the importance of interpreting the inferred parameters output from BENCH alongside these patterns of change; as the output probabilities collectively sum to 1, some probabilities may be inflated. This is especially the case for inferences relating to Nissl SAF.

#### 3.2.3 Comparison with standard model imaging

We further fitted the standard model to the data using standard model imaging (SMI) with two compartments—a stick and a zeppelin—and performed voxelwise correlations of the resulting parameters, $p_{2}$ and $f_{in}$ , with myelin-derived ODI and SAF, respectively. Our results (Supplementary Materials Fig. S3) showed fairly strong correlations between the dispersion parameter $p_{2}$ and myelin ODI (r=  -0.36, $p<10^{-4}$ ), as well as between $f_{in}$ and myelin SAF (r=  0.56, $p<10^{-4}$ ).

## 4 Discussion

In this work, we modified BENCH to relate continuous variables to biophysical parameters in highly degenerate models. We combine continuous BENCH with an extended dMRI standard model of white matter to investigate dMRI sensitivity to axons and glia. We first applied continuous BENCH to simulated data, where the parameters inferred by BENCH accurately reflected the simulated changes in spheres/cylinders in mesh substrates, validating our approach. We then applied continuous BENCH to real ex-vivo macaque MRI data with co-registered maps of microscopy metrics for myelin and cell bodies acquired from the same brain. We demonstrate that myelin SAF, a proxy for myelinated fibre density, is related to $f_{in}$ , and myelin ODI related to model parameter ODI. In addition, we found evidence that glia are poorly represented by our extended standard model. In addition to demonstrating continuous BENCH as a method for probing microstructural correlates ex-vivo, we provide a first demonstration of its application to continuous variables.

The continuous BENCH framework offers an alternative approach for linking dMRI signals to microstructural changes in the brain. BENCH’s strength lies in its ability to bypass model inversion and remain independent of specific dMRI acquisition protocols. This flexibility enables BENCH to handle highly complex biophysical models that incorporate additional parameters (e.g., cellular permeability) and to support the use of advanced dMRI acquisition protocols. Ultimately, this strength allows continuous BENCH to be applied to a wider range of MRI-microscopy studies, including those investigating neuroinflammation.

Here, we applied continuous BENCH to investigate how diffusion biophysical model parameters relate to microscopy-derived metrics. In theory, however, this approach can be used for any continuous metric. This could include continuous metrics from other MRI modalities, or non-imaging phenotypes such as blood biomarkers, heart rate, or disability scores. For example, in ongoing work, continuous BENCH has been used to explore the associations of microstructural changes with clinical phenotypes in the UK Biobank (Sudlow et al., 2015), with preliminary results linking biomarkers for diseases like hypertension and diabetes to changes in brain microstructure, such as demyelination and axonal degeneration (Kor, 2023). All this demonstrates the broad applicability of continuous BENCH across different studies and datasets to elucidate relationships between brain microstructure and any metric-of-interest.

Moreover, our trained change models can be easily applied to other datasets with similar acquisition protocols (i.e., same b-values) with minimal computational burden. Those used in this manuscript, with an extended white matter model and priors suitable for postmortem data, are made openly available (c.f. Data Availability). Trained change models based on the standard white matter model and using Human Connectome Project and UK Biobank acquisition protocols for in-vivo data are available in the original BENCH publication (Rafipoor et al., 2022).

Looking ahead, future work could also consider incorporating deep learning (DL) models into BENCH’s training phase. Instead of a generative biophysical model, a network could be used to implicitly learn relationships between microstructural changes and changes in observed dMRI data. However, such models would require a large amount of training data in which the relationship between microstructural changes and dMRI signals is well defined; without this, a DL model may offer only limited causal insights into the mechanisms driving observed changes. In contrast, as demonstrated above, the use of a generative model in BENCH aids clear interpretation by directly linking changes in microscopy to biophysical model parameters. Furthermore, training with generative models is efficient—since it involves only signal generation rather than inverse model fitting, change models only need to be trained once and can then be applied to many datasets or subjects (with data acquired using the same b-values) with minimal computational demand. Therefore, while potential efficiency gains from DL are less clear, developments in this direction warrant further investigation.

### 4.1 Numerical simulations

Our results from the numerical simulations demonstrate continuous BENCH when the ground truth is known. When applied to mesh substrates with different numbers of cylinders (representing axons) or spheres (glia soma), continuous BENCH inferred the most probable change to be an increase in $f_{in}$ and $f_{sph}$ , respectively. Cylinders are linked to a change in $f_{in}$ , accompanied by reductions in the other compartments. This may be explained by the relatively low packing densities in some substrates, where diffusing spins that encounter few membranes can be described by isotropic ($f_{ball}$ , $f_{sph}$ ), rather than anisotropic ($f_{ex}$ ) diffusion. Increasing mesh substrate spheres is linked to an increase in $f_{sph}$ , at the expense of the intra-axonal ($\Delta f_{sph}-\Delta f_{in}$ ), extra-axonal ($\Delta f_{sph}-\Delta f_{ex}$ ), and ball ($\Delta f_{sph}-\Delta f_{ball}$ ) compartment. The sphere compartment’s replacing the ball compartment may again imply the unrealistic packing densities of our substrates, as earlier suggested. These results confirm that continuous BENCH can accurately infer changes in soma- and axon-like structures, with the assumptions of impermeable membranes.

Although the SNR of 150 used in our simulations is higher than what is typically observed in ex-vivo and in-vivo data, this value was deliberately chosen to compensate for the extremely low number (i.e., 41) of substrates used in the simulation. This is because our BENCH framework bases its inference on the noise covariance matrix, which is influenced by the variance of the estimated regression coefficients β in our GLM. For our study, the variance of β (Var(β) ) is related to both the SNR in the data and the number of voxels. Mathematically, if we denote the per‐voxel noise standard deviation by σ (where $\sigma =\frac{{\text{S}}_{0}}{SNR}$ , with ${\text{S}}_{0}$ being the baseline, non-diffusion-weighted signal), and if n is the number of voxels, then the Var(β) scales as: $Var(\beta )\propto \frac{\sigma^{2}}{n}=\frac{{\left(\frac{{\text{S}}_{0}}{SNR}\right)}^{2}}{n}$. Hence, lower SNR or fewer voxels each increase the Var(β) , making BENCH’s inference less certain. Our chosen SNR of 150 in simulated data ensured that the noise covariance yielded meaningful inference despite having only 41 voxels (i.e., substrates). Given that continuous BENCH could produce meaningful inferences in this “worst-case scenario”, its performance on real datasets—with significantly more voxels—can be expected to be even more reliable. For a per-voxel SNR of 30-50, which is common for in-vivo scans, about 600 voxels for SNR = 30 and 217 voxels for SNR = 50 would be required to produce similar Var(β) . These are realistic voxel counts for real datasets. The macaque dataset investigated here used over 17,000 voxels to drive the estimates of β.

### 4.2 Ex-vivo macaque brain data

When continuous BENCH was applied to real data from an ex-vivo macaque brain, the myelin SAF, a measure of myelin density, was predominantly linked to an increasing intra-axonal compartment replacing the extra-axonal compartment ($f_{in}-f_{ex}$ ). This follows expectations, as myelinated fibres are thought to be well described by stick-like diffusion in this regime (Palombo et al., 2020; Zhang et al., 2012). Similarly, $f_{in}$ from the compressed standard model (only sticks and zeppelins) fitted via SMI correlated strongly with myelin SAF (Supplementary Materials Fig. S3), in line with previous work (Liao et al., 2024).

When considered separately, BENCH linked each microscopy-derived dispersion metric to an increased extra-axonal compartment replacing the intra-axonal compartment ($f_{ex}-f_{in}$ ). However, when considering all microscopy metrics simultaneously as regressors, the biophysical model parameter ODI was found to best explain the variation in myelin-derived ODI (Figs. 7 and 8). This confirms the model’s ODI’s specificity in mapping the dispersion of myelinated axons. The apparent relationship with the extra-axonal space (uncovered in the “single regressor” analysis) was found to be primarily driven by a strong anticorrelation between myelin SAF and ODI, and a high correlation between myelin and Nissl ODI (data not shown), highlighting the importance of accounting for the covariance in different microstructural features during MRI-microscopy comparisons. $p_{2}$—another dispersion metric from the compressed standard model (only sticks and zeppelins) fitted via SMI—also correlated well with myelin ODI (Supplementary Materials Fig. S3).

In both regression analyses—“single regressor” and “all regressors”—Nissl-derived ODI was associated with $f_{ex}\;-f_{in}$ . An increase in the extra-axonal space may be associated with fibre geometries that are more dispersed and therefore less tightly packed. Hence, our results may suggest Nissl-derived ODI as an indirect measure of fibre dispersion, albeit less specific than myelin-derived ODI. Nissl orientations are derived via a structure tensor which outputs orientations similar to those from myelin stains. This similarity may arise because glial cells cluster in the spaces between axons, creating an apparent orientation that mirrors the axonal orientation (Schurr & Mezer, 2021). However, exactly how we should interpret these orientations requires more investigation, where there may be important differences between myelin- and Nissl-derived ODI (i.e., two metrics are not equivalent). Notably here, the myelin ODI was linked to the standard model ODI parameter, whilst the Nissl ODI was not. This warns against their interpretation as being equivalent measures of microstructure dispersion.

Interpreting the Nissl SAF is more challenging. Nissl stains the cell soma of both neurons and glia indiscriminately. Since there are very few neuronal soma in WM, our Nissl SAF likely reflects glial cell soma density. This serves as a proxy for glia cell density, meaning our correlations could pick up on MRI sensitivity to glia cell bodies themselves or glia cells as a whole (soma and processes). There are multiple ways in which glia may contribute to the dMRI signal. The cell bodies could behave like impermeable spheres (changes in $f_{sph}$ , $D_{sph}$ , $R_{sph}$ ), the processes could contribute to anisotropy (changes in ODI), and/or the glia could be in fast exchange with, and thus assimilated into, the extracellular space (changes in $f_{ex}$ ). Investigation of the dMRI patterns of change (Fig. 9, all regressors) revealed that none of the change models’ predicted patterns of change adequately matched that from the Nissl SAF. In this case, BENCH infers the parameter with the highest probability (here $f_{ex}\;-f_{sph}$ ), although it may not be a good fit and the probability (normalised across all parameters) may be inflated. The lack of a match suggests that no single parameter from the extended standard model, in its current form, is capable of describing signal variation due to Nissl SAF. Modelling glia simply as impermeable spheres does not produce a good match, nor does assuming fast exchange with the extracellular space, as changes in $f_{ex}$ also failed to match the observed pattern of change well. The Nissl SAF pattern of change (Fig. 9, left) shows changes in both the mean and l2 dMRI summary measures. Figure 9 (right) shows how changes in the $b_{7}$ and $b_{10}$ mean summary measures are likely driven by changes in compartment signal fractions (i.e., $f_{in}$ , $f_{ex}$ , $f_{sph}$ ), and the l2 terms (with $b_{10}\;l2>b_{7}\;l2$ ) are related to signal anisotropy and are most often driven by changes in dispersion (ODI or $f_{in}$ ). It is, therefore, possible that a better match may be found if BENCH were to consider both the ODI and compartment fractions changing together (more glia and more anisotropy). This potentially suggests that both glia cell bodies and processes contribute meaningfully to the signal. We try to account for the effects of glia-related anisotropy through Nissl ODI. However, as Nissl ODI is calculated from a stain of the glia soma rather than the processes, it may not accurately reflect the anisotropy of the of the glia processes. Consequently, we conclude that the representation of glia in BENCH could be improved by incorporating more complex biophysical models (e.g., with exchange or inter-compartmental variations in $T_{2}$), or more complex change models (e.g., with changes in more than two microstructure compartments at the same time, or the simultaneous change of a signal fraction and ODI). Nonetheless, our results show that there are substantial changes in the dMRI summary measures with respect to Nissl SAF, implying non-negligible dMRI signal sensitivity to changes in glial cell soma density (or other correlated features), after accounting for myelin load and dispersion. This finding is in agreement with recent evidence of dMRI’s sensitivity to glia density (Garcia-Hernandez et al., 2022; Guglielmetti et al., 2016; Kor et al., 2022; Taquet et al., 2019; Zhuo et al., 2012).

In this study, we applied the continuous BENCH framework to the extended WM standard model as a proof-of-concept, showcasing its viability for application to highly complex biophysical models. For comparison with existing methods and to further validate our results, we also performed voxelwise correlations between SMI parameters and microscopy-derived metrics (Supplementary Materials Fig. S3). Note that SMI only considered a compressed standard model with sticks and zeppelins (6 free parameters), while BENCH considered an extended standard model with sticks, zeppelins, dot, sphere (and optional ball) compartments (9-11 free parameters). Both SMI and BENCH similarly related dispersion parameters ($p_{2}$ and ODI) to myelin ODI, and $f_{in}$ to myelin SAF, giving confidence to both results. The advantage of using a model like BENCH over SMI is that it allows for the inclusion of extended, otherwise degenerate models with additional compartments—such as glia—that cannot be reliably estimated from two-shell HARDI data. SMI fits parameters from rotational invariants of the signal. When considering data with two shells each with three rotational invariants, we are limited to six observations, and thus can only estimate up to six parameters. In contrast, BENCH can infer parameter changes even in highly degenerate models with many more free parameters, offering greater flexibility to include additional tissue compartments and potentially enabling improved microstructural interpretation.

In summary, this study establishes a proof-of-concept framework for modelling microstructural changes in ex-vivo macaque data where MRI and microscopy are available. As the microstructural building blocks of the brain are largely conserved across mammals, with relatively subtle changes to glia and neuron morphologies, the findings are likely not species-specific. Our study contributes to literature validating the standard model ODI parameter for investigation of fibre dispersion, and $f_{in}$ relating to the amount of myelinated fibres. Further, our results suggest sensitivity of dMRI to glia density, which could be confirmed in human tissues. This is important as investigations into the ability of in-vivo dMRI to detect glia changes in in-vivo human data could provide new opportunities for imaging glia-related pathologies and neuroinflammation.

### 4.3 Limitations

There are limitations to this study. First, continuous BENCH assumes change in only one (e.g., diffusivities, ODI) or two (e.g., signal fractions) parameters. However, it is possible that simultaneous changes in more than two parameters may better explain the histological changes in pathology. Future work with BENCH includes expanding this framework to accommodate the simultaneous change of more than two parameters, as demonstrated in appendix A of Rafipoor et al. (2022). Second, our numerical simulation only validates continuous BENCH’s ability to accurately infer simple changes in intra-soma and intra-axonal compartments in specific conditions, such as the case where membranes are impermeable. These conditions may not be met when applying continuous BENCH to ex-vivo data, where some assume glia to be in fast exchange with the extra-axonal space (Badaut et al., 2011; Nilsson et al., 2013), though exchange is likely attributed to glial processes rather than soma (Palombo et al., 2020). Third, our study is limited by the use of a linear diffusion encoding protocol with two shells, which restricts sensitivity to certain model parameters, particularly compartmental diffusivities (Liao et al., 2024). This protocol was chosen to match the ex-vivo macaque brain data that had been acquired prior to this study with a HARDI acquisition (i.e., linear encoding, multi-shell data) as is typical in many other datasets. However, our framework allows flexibility in diffusion protocols. Therefore, future work could explore more advanced encoding schemes (e.g., planar or spherical) to better estimate and link these model parameters to microscopy-derived metrics. Fourth, the mesh substrates used for simulations are not very biologically realistic. However, these substrates were designed to enable systematic parameterisation of compartments, such as axon radius, glial soma size, and packing density. This approach allows for controlled and reproducible simulations, but does not fully capture the complexity of biological tissue. Future work could include simulations on more refined synthetic meshes to include more realistic packing densities, cellular morphologies (Callaghan et al., 2020; Ginsburger et al., 2019; Palombo et al., 2019; Villarreal-Haro et al., 2023), and axon diameters (e.g., 1–2 μm ; Veraart et al., 2020), bridging the gap between computational feasibility and biological realism. Furthermore, ultra-realistic 3DEM-derived reconstructions could also be employed to validate the continuous BENCH framework. Fifth, we do not incorporate other microscopy-derived metrics that may relate to dMRI. For example, we do not include metrics derived from neurofilament staining, which may better explain variation in intra-axonal signal fraction and/or ODI, as our myelin metrics may miss contributions from low/unmyelinated axons.

## Data and Code Availability

The BigMac dataset is openly available via the Digital Brain Bank: https://open.win.ox.ac.uk/DigitalBrainBank/. The code and the trained change models are available at https://github.com/danzlkor/continuous-bench.

## Author Contributions

D.Z.L.K. conceived the study design and implemented continuous BENCH, which involved adapting the code from the original BENCH framework. D.Z.L.K. also conducted data simulation, data analyses, and drafted the manuscript. H.R. modified the BENCH framework and helped interpret results from data analyses. I.N.H. developed the TIRL registration platform and performed the MRI-microscopy co-registration. A.S. and G.D. processed and curated microscopy data. S.J. conceived the study design and contributed to the design of continuous BENCH. M.C. contributed to the design of continuous BENCH and helped interpret results from data analyses. K.L.M. conceived the study design, contributed to all data analyses, and helped draft the manuscript. AFDH conceived the study design, contributed to implementation of continuous BENCH, contributed to all data analysis, and helped draft the manuscript.

## Ethics Declaration

This study analysed postmortem data in the macaque brain. No specific ethics were required for the acquisition and analysis of these data.

## Declaration of Competing Interest

None.

## Supplementary Material

Supplementary Material
