# Supplementary material for: Linking microscopy to diffusion MRI with degenerate biophysical models: An application of the Bayesian EstimatioN of CHange (BENCH) framework
Source: Imaging Neurosci (Camb). 2025 Jul 24;3:IMAG.a.85. doi: 10.1162/IMAG.a.85 (PMC12330835; doi:10.1162/IMAG.a.85)
Supplement: Supplementary Material [file IMAG.a.85_supp.pdf]

Supplementary Materials for "Linking microscopy to diffusion MRI with degenerate biophysical models: an application of the Bayesian EstimatioN of CHange (BENCH) framework"

Theory of BENCH

Details of BENCH have been previously described in (Rafipoor et al., 2022). In BENCH, we bypass model inversion by instead inferring $P(\hat{\boldsymbol{\nu}}|\mathbf{y}, \mathbf{\Delta y})$, the probability of a change in given biophysical parameter ($\hat{\boldsymbol{\nu}}$) given a baseline dMRI signal ($\mathbf{y}$) and a measured change in dMRI signal ($\mathbf{\Delta y}$). This process is formalised using Bayes' rule:

$$P(\hat{\boldsymbol{\nu}} \mid \mathbf{y}, \mathbf{\Delta y}) = \frac{P(\mathbf{\Delta y} \mid \mathbf{y}, \hat{\boldsymbol{\nu}}) P(\hat{\boldsymbol{\nu}} \mid \mathbf{y})}{P(\mathbf{\Delta y} \mid \mathbf{y})}, \tag{1}$$

where the denominator is a normalisation constant (often called the evidence), and $P(\hat{\boldsymbol{\nu}} \mid \mathbf{y})$ is the prior on the parametric change $\hat{\boldsymbol{\nu}}$. In principle, the prior can be any form of distribution. We assumed a uniform distribution, which implies that no specific change is more likely than any other, given any measured baseline dMRI signal. Hence, we only need to estimate the likelihood $P(\mathbf{\Delta y}|\mathbf{y}, \hat{\boldsymbol{\nu}})$. This can be achieved by first computing:

$$P(\mathbf{\Delta y}|\mathbf{y}, \hat{\boldsymbol{\nu}}, |\Delta \nu|) = N(|\Delta \nu| \boldsymbol{\mu}(\mathbf{y}), |\Delta \nu|^2 \boldsymbol{\sigma}(\mathbf{y}) + \boldsymbol{\sigma_l}), \tag{2}$$

where $|\Delta \nu|$ is the magnitude of change for a change in parameter set $\boldsymbol{\nu}$ of arbitrary size:

$$|\Delta \nu| = \frac{\boldsymbol{\Delta \nu}}{\hat{\boldsymbol{\nu}}}. \tag{3}$$

N is a normal distribution, with mean that describes the spread in of possible change vectors originating from some baseline signal $\mathbf{y}$ (Figure 2). $\boldsymbol{\sigma_l}$ is the noise covariance matrix of the continuous variable (indexed l) as estimated from the data (c.f. Section 2.3).

Since we only aim to identify if a parameter is changing, rather than the magnitude of that change, we marginalise $P(\Delta \mathbf{y}|\mathbf{y}, \hat{\boldsymbol{\nu}}, |\Delta \nu|)$ with respect to $|\Delta \nu|$ to get $P(\mathbf{\Delta y}|\mathbf{y}, \hat{\boldsymbol{\nu}})$. We estimate $\boldsymbol{\mu}(\mathbf{y})$ and $\boldsymbol{\sigma}(\mathbf{y})$ using trained change models, as outlined in Section 2.1.2. Please refer to (Rafipoor et al., 2022) for detailed derivations.

Training regression models to estimate distribution of change vectors

For $\mu_i(S)$, the regression model is:

$$\boldsymbol{\mu}_i(\mathbf{S}) = \mathbf{S} \cdot \mathbf{F} \cdot \boldsymbol{\omega}_i, \tag{4}$$

where $\mathbf{S} \cdot \mathbf{F}$ is the design matrix. $\mathbf{F}$ is a linear transformation of $\mathbf{S}$, where $\mathbf{S} \cdot \mathbf{F}$ produces a basis set that can characterise $\boldsymbol{\mu}_i(\mathbf{S})$ varies over $\mathbf{S}$. ω_i are the regression weights, with dimensions defined by the number of variables in the basis set (rows) and the number of dMRI summary measures (columns).

$\boldsymbol{\sigma}_i(\mathbf{S})$ is estimated with a similar formulation, albeit with an additional transformation T to ensure that $\boldsymbol{\sigma}_i(\mathbf{S})$ is a positive definite matrix:

$$\boldsymbol{\sigma}_i(\mathbf{S}) = \mathbf{T} \cdot (\mathbf{S} \cdot \mathbf{G} \cdot \boldsymbol{\eta}_i). \tag{5}$$

$\mathbf{G}$ is another linear transformation. In order to account for the positive definite nature of $\boldsymbol{\sigma}_i(\mathbf{S})$, $\mathbf{T}$ includes the arrangements of elements, exponentiation of the diagonals and the matrix multiplication for the inverse Cholesky decomposition. η_i are the weights , with the row length set by the number of independent variables and the number of columns corresponding to the unique elements in the covariance matrix $\boldsymbol{\sigma}_i$.

Inserting these regression models into the normal distribution $N(\boldsymbol{\mu}_i(\mathbf{S}), \boldsymbol{\sigma}_i(\mathbf{S}))$:

$$J = N\big(\mathbf{S} \cdot \mathbf{F} \cdot \boldsymbol{\omega}_i, \mathbf{T} \cdot (\mathbf{S} \cdot \mathbf{G} \cdot \boldsymbol{\eta}_i)\big), \tag{6}$$

where the likelihood of observing $\frac{\partial \mathbf{S}}{\partial \hat{\nu}_i}$ is:

$$L(\boldsymbol{\omega}_i, \boldsymbol{\eta}_i) = \prod_r J\left(\frac{\partial \mathbf{S}}{\partial \hat{\boldsymbol{v}}_i}, \mathbf{S}\right). \tag{7}$$

The optimal weights ω_i,η_i are estimated using a maximum likelihood optimisation and r pairs of ($\frac{\partial \mathbf{S}}{\partial \hat{\nu}_i}$,$\mathbf{S}$) from the training dataset. These weights can then be used to estimate $\boldsymbol{\mu}_i$ and $\boldsymbol{\sigma}_i$ at any observed baseline dMRI signal $\mathbf{y}$ derived from real data via Equations 4 and 5 (in Appendix B), respectively.

Summary measures for diffusion MRI data

dMRI summary measures were calculated using the spherical harmonic decomposition (Tournier et al., 2004) of the dMRI signal. Here, the dMRI signal can be decomposed into a unique linear combination of orthonormal basis functions over the surface of a unit sphere (spherical harmonics):

$$S(\theta,\phi)=\sum_{l=0}^{\infty}\sum_{m=-l}^{l}C_{l,m}Y_{l,m}(\theta,\phi). \tag{8}$$

The dMRI signal S, parameterised by the polar (θ) and azimuthal (ϕ) angles of the standard spherical coordinate system, is now described as a linear combination of coefficients (C) and spherical harmonics (Y) up to a pre-decided degree $l=0,1,2,...$ and order $m=-l,...,l$. Since S is symmetric around the origin, only even-degree harmonics (i.e. $l=0,2,4,...$) have non-zero coefficients.

We then computed rotationally invariant measures for each shell of dMRI signal as:

$$y_l=\frac{1}{2l+1}\sum_{m=-l}^{l}C_{l,m}^2, \tag{9}$$

where y_l is the summary measure at degree l for a single shell of data (Kazhdan et al., 2003; Novikov et al., 2018). Here, we set $l=0,2$ (i.e. 0th and 2nd degree), since a higher l quantifies the signal anisotropy, but at a much lower SNR. Lastly, we took the mean over the norm to ensure that the scale remains constant across degrees. With this formulation, we summarised all measured dMRI signal data points with 4 rotationally invariant summary measures (2 summary measures $\times$ 2 shells) per voxel.

Mask for ex-vivo macaque brain data

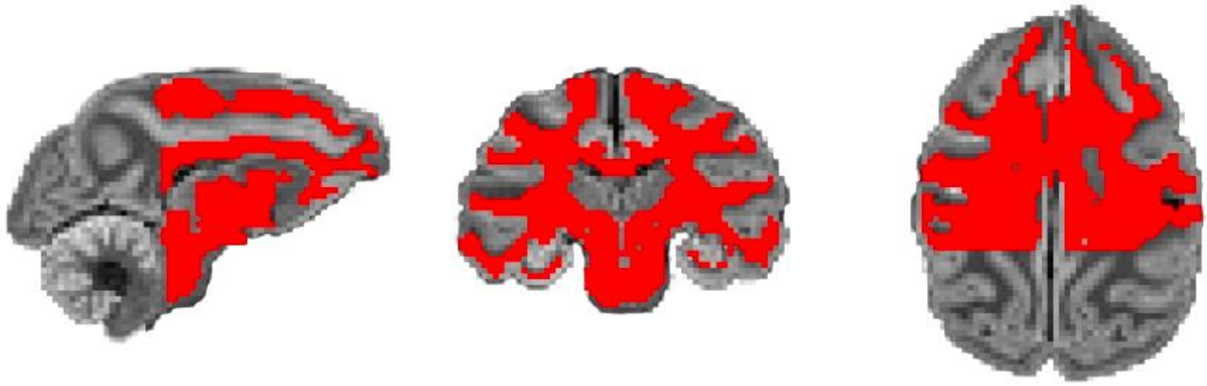

Figure S1: The final WM mask (red) containing the 17945 voxels used for analysis. The mask is overlaid on the ex-vivo dMRI data of the macaque brain.

Voxelwise correlations between dMRI summary measures and microscopy-derived metrics for ex-vivo macaque brain data

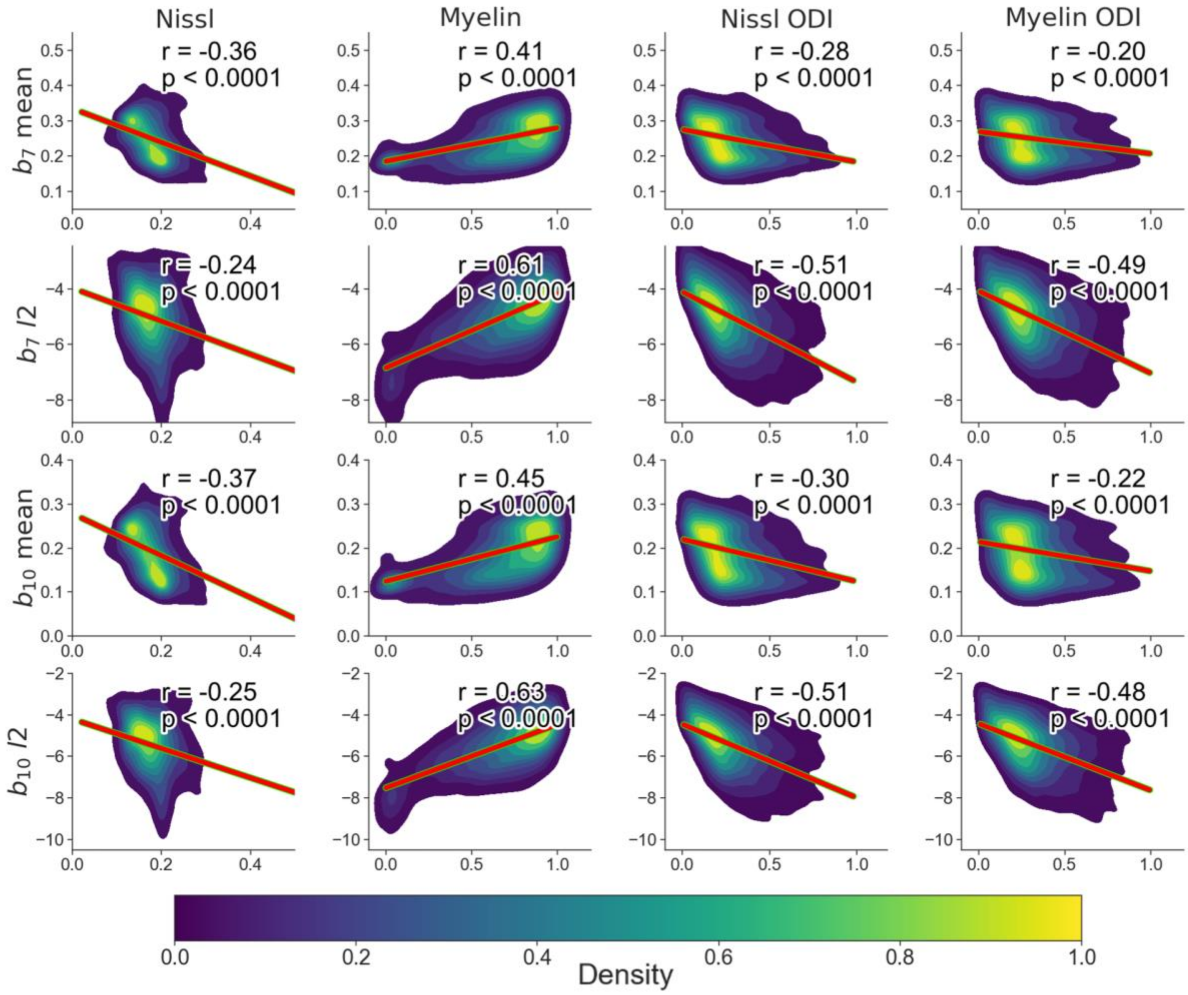

Figure S2: Voxelwise correlations between the dMRI measures (rows; b_7 mean, b_7 $l2$, b_{10} mean, b_{10} $l2$) and the four microscopy-derived metrics (columns; Nissl, Myelin, Nissl ODI, Myelin ODI) across 17,945 voxels in ex-vivo macaque white matter. Each panel shows a 2D density plot of the joint distribution. The best-fit slope from the linear regression (red) is shown, along with the Pearson correlation coefficient (r) and p-values. These correlations highlight the dynamic range of both dMRI and microscopy-derived measures across the voxels, and how the dMRI summary measures vary in relation to the microscopy-derived metrics.

Voxelwise correlations between standard model imaging parameters and myelin-derived metrics for ex-vivo macaque brain data

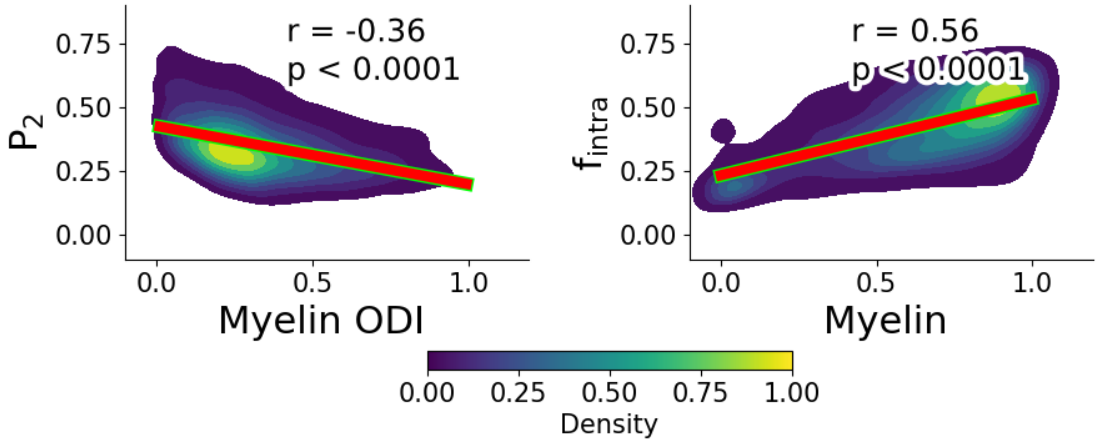

Figure S3: Voxelwise correlations between standard model imaging (SMI) parameters and myelin-derived metrics. The standard model with two compartments—a stick and zeppelin—were fitted voxelwise to the data using the SMI toolbox (Novikov et al., 2018; Reisert et al., 2017; Coelho et al., 2022), and the output parameters correlated against histology in the white matter. Left: The second-order spherical harmonic coefficient p_2 is correlated with microscopy-derived myelin ODI ($r = -0.36,\ p < 10^{-4}$). Right: SMI's intra-axonal volume fraction (f_{in}) is correlated with microscopy-derived myelin density ($r = 0.56,\ p < 10^{-4}$). Background density illustrates voxel counts (violet = low density; yellow = high density), and the red line is the best-fit slope from the linear regression.
